# Identification of *RNF213* as a Susceptibility Gene for Moyamoya Disease and Its Possible Role in Vascular Development

**DOI:** 10.1371/journal.pone.0022542

**Published:** 2011-07-20

**Authors:** Wanyang Liu, Daisuke Morito, Seiji Takashima, Yohei Mineharu, Hatasu Kobayashi, Toshiaki Hitomi, Hirokuni Hashikata, Norio Matsuura, Satoru Yamazaki, Atsushi Toyoda, Ken-ichiro Kikuta, Yasushi Takagi, Kouji H. Harada, Asao Fujiyama, Roman Herzig, Boris Krischek, Liping Zou, Jeong Eun Kim, Masafumi Kitakaze, Susumu Miyamoto, Kazuhiro Nagata, Nobuo Hashimoto, Akio Koizumi

**Affiliations:** 1 Department of Health and Environmental Sciences, Kyoto University, Kyoto, Japan; 2 Faculty of Life Sciences, Kyoto Sangyo University, Kyoto, Japan; 3 Department of Molecular Cardiology, Osaka University, Suita, Osaka, Japan; 4 Department of Neurosurgery, Kyoto University, Kyoto, Japan; 5 National Cerebral and Cardiovascular Center, Suita, Osaka, Japan; 6 Comparative Genomics Laboratory, National Institute of Genetics, Mishima, Shizuoka, Japan; 7 Principles of Informatics Research Division, National Institute of Informatics, Tokyo, Japan; 8 Palacky University Hospital, Olomouc, Czech Republic; 9 Department of Neurosurgery, University of Tubingen, Tubingen, Germany; 10 Department of Pediatrics, Chinese People's Liberation Army General Hospital, Beijing, People's Republic of China; 11 Seoul National University College of Medicine, Seoul, Korea; Charité Universitaetsmedizin Berlin, Germany

## Abstract

**Background:**

Moyamoya disease is an idiopathic vascular disorder of intracranial arteries. Its susceptibility locus has been mapped to 17q25.3 in Japanese families, but the susceptibility gene is unknown.

**Methodology/Principal Findings:**

Genome-wide linkage analysis in eight three-generation families with moyamoya disease revealed linkage to 17q25.3 (*P*<10^-4^). Fine mapping demonstrated a 1.5-Mb disease locus bounded by D17S1806 and rs2280147. We conducted exome analysis of the eight index cases in these families, with results filtered through Ng criteria. There was a variant of p.N321S in *PCMTD1* and p.R4810K in *RNF213* in the 1.5-Mb locus of the eight index cases. The p.N321S variant in *PCMTD1* could not be confirmed by the Sanger method. Sequencing *RNF213* in 42 index cases confirmed p.R4810K and revealed it to be the only unregistered variant. Genotyping 39 SNPs around *RNF213* revealed a founder haplotype transmitted in 42 families. Sequencing the 260-kb region covering the founder haplotype in one index case did not show any coding variants except p.R4810K. A case-control study demonstrated strong association of p.R4810K with moyamoya disease in East Asian populations (251 cases and 707 controls) with an odds ratio of 111.8 (*P* = 10^−119^). Sequencing of *RNF213* in East Asian cases revealed additional novel variants: p.D4863N, p.E4950D, p.A5021V, p.D5160E, and p.E5176G. Among Caucasian cases, variants p.N3962D, p.D4013N, p.R4062Q and p.P4608S were identified. *RNF213* encodes a 591-kDa cytosolic protein that possesses two functional domains: a Walker motif and a RING finger domain. These exhibit ATPase and ubiquitin ligase activities. Although the mutant alleles (p.R4810K or p.D4013N in the RING domain) did not affect transcription levels or ubiquitination activity, knockdown of *RNF213* in zebrafish caused irregular wall formation in trunk arteries and abnormal sprouting vessels.

**Conclusions/Significance:**

We provide evidence suggesting, for the first time, the involvement of *RNF213* in genetic susceptibility to moyamoya disease.

## Introduction

**Figure 1:**
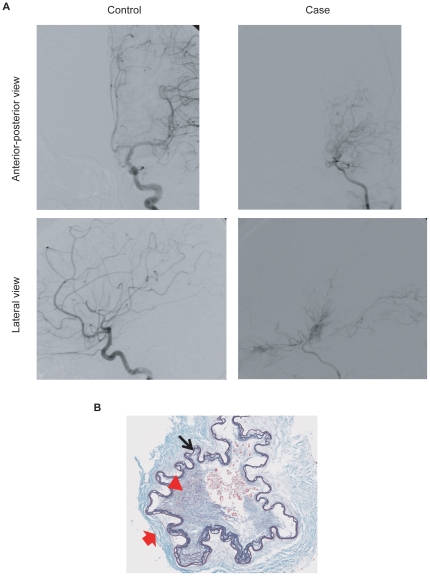
Clinical features of moyamoya disease. (**A**) An anterior-posterior and lateral views of left internal carotid angiography in a 9-year-old child with moyamoya disease (right) and an 8-year-old control child (left). An occlusion with moyamoya vessels can be seen around the terminal portion of the internal carotid artery and proximal portions of the anterior cerebral artery and middle cerebral artery in the affected child. (**B**) Intimal hyperplasia in the middle cerebral artery from an autopsy of a 69-year-old case. Intimal hyperplasia (red arrowhead), atrophic media (red arrow) and winding internal elastic lamina (black arrow) can be seen with Elastica Masson staining, ×200.

Moyamoya disease is an idiopathic disorder characterized by occlusive lesions in the supraclinoid internal carotid artery and its main branches in the circle of Willis. To compensate for the blood flow around the occlusive region, a fine vascular network develops that resembles “puffs of smoke” (Figure 1A) [1]. The unique appearance of moyamoya vessels described by Suzuki and Takaku in 1969 [2] spurred international recognition of moyamoya disease [MIM 607151] (Online Mendelian Inheritance in Man in Appendix S1). Moyamoya disease occurs worldwide [3], but its prevalence is highest in East Asian countries, including Japan (1 in 10,000), Korea and China [4], [5]. It is known to cause stroke in neonates and children, and therefore pathological clues for early diagnosis and novel therapeutic approaches are needed [6].

The occlusive lesions are caused by excessive proliferation of smooth muscle cells (SMCs), which specifically occurs in arteries in the skull with compensatory bypass vessels (Figure 1B) [7]. These occlusive lesions are similar to those of atherosclerosis in that both types of lesion exhibit endothelial injury, SMC proliferation and intimal hyperplasia. However, there are major differences between the two types: in moyamoya vessels, infiltrating cellular components such as macrophages and lipid deposits are not observed [8]. A recent study revealed that accelerated vascular remodeling might underlie the pathological consequences of moyamoya disease [9]. Clinical investigations have revealed elevated levels of certain growth factors or cytokines in the cerebrospinal fluid, serum or blood vessels of patients with moyamoya disease [10]–[15]. These factors are assumed to be associated with vascular remodeling.

Epidemiological studies have revealed several risk factors associated with moyamoya disease, including Asian ethnicity, female gender and a family history of the disease [4]. Given that 15% of moyamoya patients have a family history of the disease, genetic factors are suspected to underlie its pathogenesis.

Several studies have explored genetic factors and revealed four loci associated with moyamoya disease: 3p24–p26 [16], 6q25 [17], 8q23 [18] and 17q25 [19]. The locus on 17q25, first mapped by Yamauchi *et al*. [19], was replicated in 2008 [20], [21]. In our previous study, a strong association of a variant (ss161110142) was demonstrated in East Asian patients, suggesting a possible East Asian founder haplotype for moyamoya disease [21]. The major aim of the present study was to identify the causative gene for moyamoya disease. In the present study, we conducted exome analysis and identified ring finger protein 213 (*RNF213*; DDBJ/EMBL/GenBank accession number AB537889) [National Center for Biotechnology Information (NCBI) in Appendix S1] as a susceptibility gene. In this study, we cloned *RNF213* and determined its open reading frame (ORF). Compared with the previously registered *RNF213* sequence [NM_020914.4] (NCBI in Appendix S1), our *RNF213* clone possesses a 2,500-bp longer 3′ untranslated region (UTR) and lacks exon 4. Since we found that AB537889 seems to be the major transcript of *RNF213*, the descriptions of *RNF213* in this paper are primarily based on the newly determined ORF unless otherwise stated.

## Methods

### Ethical statement

Ethical approval for this study was given by the Institutional Review Board and Ethics Committee of Kyoto University School of Medicine, Kyoto University, Japan (approval number: G140; approval date: 10/18/2004); by the Ethics Committee of the medical faculty of the University of Tübingen (Ethik-Kommission der Medizinischen Fakultät des Universitätsklinikums Tübingen; permit number: 273/2009BO1; approval date: 1/1/2009); by Seoul National University Hospital Institutional Review Board (approval number: H0507-509-153; approval date 8/24/2005); by the Medical Ethics Committee of Beijing Children's Hospital Institutional Review Board, Capital Medical University, China (approval number: 2005-31; approval date: 3/15/2005); and by the Ethics Committee of University Hospital and the Faculty of Medicine of Palacky University in Olomouc (reference number: 62/10; approval date: 8/18/2008). Participants were recruited in these institutes. All subjects gave written informed consent, or for those considered too young to consent, informed consent was given by their parent or guardian.

### Subjects

Diagnostic criteria were based on the Japanese Research Committee on moyamoya disease of the Ministry of Health, Welfare and Labour, Japan (RCMJ) criteria [22]. Information on family histories, gender, age of onset, onset of symptoms and unilateral or bilateral moyamoya disease was obtained either by interview or by clinical chart review, as previously reported [21], [23].

We recruited East Asian cases (Japanese, Korean and Chinese), as described previously [21]. The participants comprised 41 Japanese families and one Korean family (Figure S1 and Table S1), 209 unrelated cases (120 Japanese, 37 Korean and 52 Chinese). We recruited 757 unrelated controls: 384 Japanese, 223 Korean and 100 Chinese (Table S2) and an additional 50 Chinese adult females [Mean age at participation (years ± SD): 38.4±10.5].

We also recruited a Caucasian family of Czech ethnicity (Figure S2A) and 7 unrelated Czech and 42 German cases with moyamoya disease at the University of Tubingen (Germany), and Palacky University and University Hospital (Olomouc, Czech Republic). The mean age (years ± SD) at diagnosis was 30.4±14.7, and the sex ratio of males:females was 16∶33. The 284 Caucasian controls were recruited from Palacky University and University Hospital (*n* = 120), the University of Tubingen (*n* = 164) and another 100 Caucasian control samples (the Coriell Caucasian Panel) were bought from the Coriell Institute. The mean age (years ± SD) was 43.9±17.9 and the sex ratio of males:females was 176∶208. No magnetic resonance imaging screening of the controls was conducted.

### Linkage, haplotype and segregation analyses

**Figure 2:**
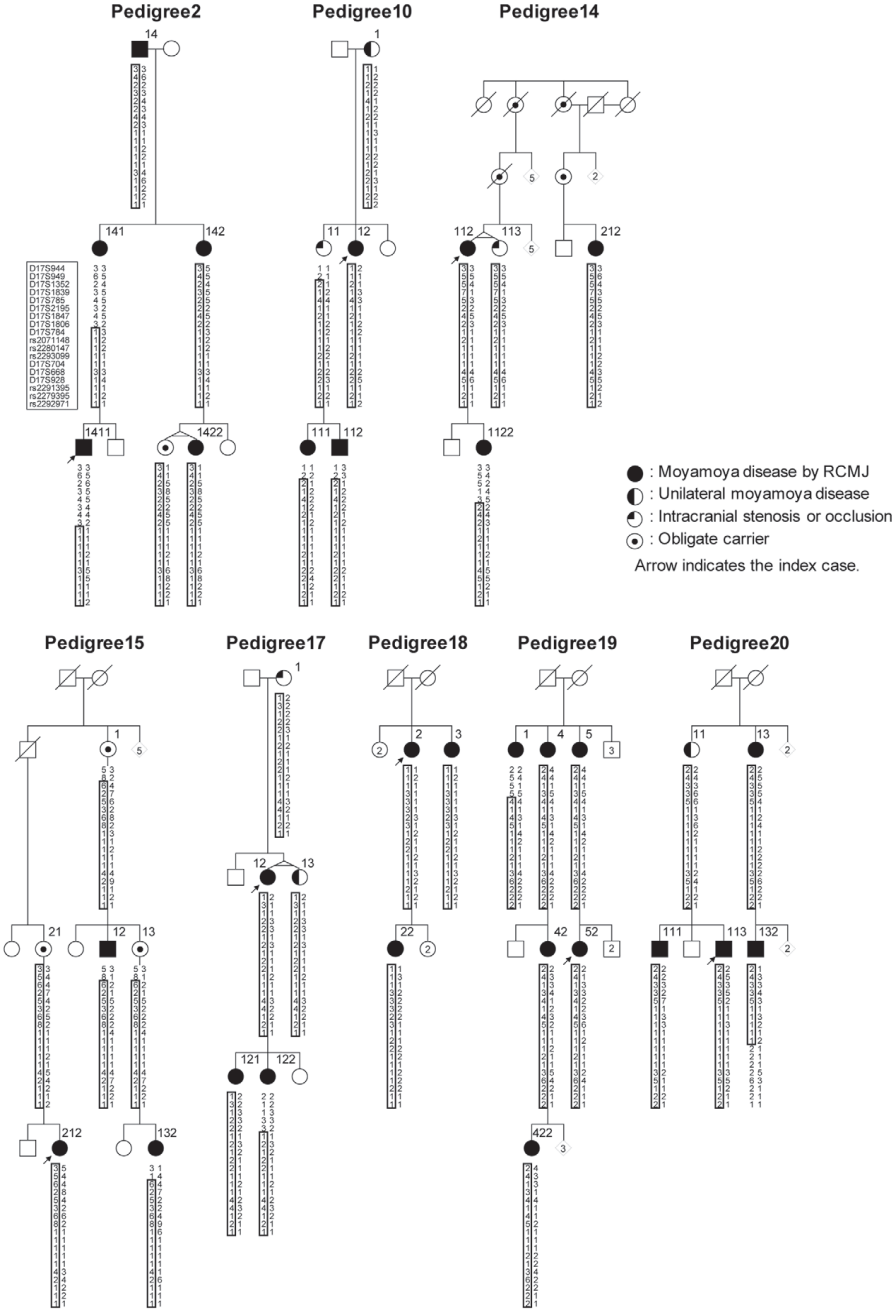
The eight three-generation families and haplotype construction with fine markers. For familial index cases in the eight pedigrees, the haplotypes are indicated by the boxes constructed in GENEHUNTER [24]. The smallest disease haplotype spanning from D17S1806 to rs2280147 was estimated by the region common to both 1411 in the pedigree 2 and 132 in pedigree 20.

We conducted a parametric genome-wide linkage analysis using GENEHUNTER [24] (GENEHUNTER Ver2.1_r6: Appendix S1) in the eight largest three-generation families (peds. 2, 10, 14, 15, 17–20) (Figure 2) with an ABI Prism Linkage Mapping Set (Version 2; Applied Biosystems) with 382 markers, 10 cM apart, for 22 autosomes, and fine-mapping markers designed according to information from the uniSTS reference physical map (http://www.ncbi.nlm.nih.gov/genome/sts/). Unaffected members were treated as “phenotype unknown” because of the incomplete and age-dependent penetrance of moyamoya disease [20]. We considered obligate carriers and subjects with unilateral occlusion or stenosis around the circle of Willis as affected, as previously reported [20]. The disease allele frequency was set at 0.00002 and a phenocopy frequency of 0.000001 was assumed, as previously reported [20]. Population allele frequencies were assigned equal portions for individual alleles. We performed multipoint analyses for autosomes and obtained logarithm of the odds (LOD) scores. Haplotypes were constructed with GENEHUNTER and segregation was investigated using the constructed haplotypes.

To calculate the statistical significance level of the linkage to 17q25.3 in the eight families, we applied a bootstrap method using simulation, as previously reported [25]. We simulated recombination events for 22 chromosomes in these eight families, calculated multipoint LOD scores by GENEHUNTER and obtained the maximum total LOD scores in each trial. The simulation was run 10,000 times.

### Exome analysis

We conducted exome analysis on one index case from each of the eight families (peds. 2, 10, 14, 15, 17–20) (Figure 2), who were diagnosed with moyamoya disease based on RCMJ criteria. We targeted all protein-coding regions as defined by RefSeq37 (RefSeq in Appendix S1). Approximately 210,121 coding exons in the 20,794 target genes from 10μg of genomic DNA from each case were captured using an Agilent SureSelect Human All Exon kit, following the manufacturer's protocols. Briefly, DNA was sheared by acoustic fragmentation (Covaris) and purified using a QIAquick PCR Purification kit (Qiagen). The quality of the fragmentation and purification was assessed with an Agilent 2100 Bioanalyzer. The fragment ends were repaired and adaptors were ligated to them (NEBNext DNA sample prep; New England Biolabs). The resulting DNA library was purified using a QIAquick PCR Purification kit, amplified by PCR and captured by hybridization to the biotinylated RNA library “baits” (Agilent). The whole-exome DNA library was sequenced on an Illumina Genome Analyzer IIx in 76-bp paired-end reads using two channels. Sequence reads were mapped to the reference human genome (Ghr37/hg19) (NCBI and UCSC Genome Browser in Appendix S1) using ELANDv2 software (Illumina). Variant detection was performed with CASAVA software (version 1.7; Illumina). Sequence calls were filtered to include only those with >8 coverage, >30 consensus quality and >20 mapping quality. Candidate variants were filtered by SAMtools (Appendix S1). For comparison, we used an exome database of five Japanese controls, who do not have histories of stroke, for which the analysis was conducted by the same protocol and experimental procedures.

### Direct sequencing of *RNF213* and *PCMTD1* by the Sanger method

Genomic DNA was extracted from blood samples with a QIAamp DNA Blood Mini kit (Qiagen). The whole *RNF213* gene was sequenced, including the 5′ promoter region at least 2 kb upstream of the first exon, 50–300 bp of each intron upstream and downstream of each exon, all the exons and throughout the 3′ UTR. Target sequences, including the skipped exon 4 were based on the open reading frame of NM_020914.4. Sequencing exon 8 of *PCTDM1* was conducted to confirm the exome result. Sequencing primers were commercially synthesized (PROLIGO Primers & Probes) (Table S3) (Appendix S1). The PCR products were separated on an ABI Prism 3130 Avant DNA sequencer (Applied Biosystems, Tokyo, Japan).

### Deep sequencing of introns and intergenic regions of *RNF213*

To search for a hidden causative variant, we sequenced the entire 260-kb genomic region from the 5′ end of *SLC26A11* (bp 78194200) to the 5′ end of *NPTX1* (bp 78450404) (NCBI in Appendix S1) in the index case of pedigree 11, who was homozygous at 17q25.3 because of parental consanguineous marriage, using two bacterial artificial chromosome (BAC) clones (bp 78194200–78238718 and bp 78345170–78450404) (Table S4) or by direct sequencing. The entire RNF213 gene including promoters, 5′UTR, introns, exons and 3′UTR was sequenced in a control sample in the same way as for the index case of pedigree 11. Since we failed to sequence intron 15 of *RNF213*, we used Southern blotting to detect a possible expansion. Details of the BAC cloning and Southern blotting are described in the Text S1. The primer sequences are listed in Table S3.

### Genotyping of five rare variants (ss179362671, ss179362673 (p.R4810K), ss179362674, ss179362675 ss161110142) and 34 other single nucleotide polymorphisms (SNPs)

Typing of the five rare variants and 34 SNPs was conducted using Taqman probes (TaqMan SNP Genotyping Assays; Applied Biosystems) using a 7300/7500 Real-Time PCR System (Applied Biosystems) (dbSNP 131 and Hapmap database in Appendix S1). The transmitted haplotypes in 42 families were constructed using GENEHUNTER. Details of the 39 SNPs are described in the Text S1. The allele frequencies were determined in Japanese controls.

### Copy number variations (CNVs)

We performed genotyping with Affymetrix 500K arrays (GeneChip® Mapping 250/500 K; Affymetrix, Inc.) according to the manufacturer's protocol. Genomic DNA samples donated by the index cases of pedigrees 5, 11 and 18 and the control spouse of 2 of pedigree 18 were analyzed. The genome-wide call rates were >95% for all the genomic DNAs. CNVs were identified from the intensities using the Affymetrix GeneChip® Chromosome Copy Number Analysis Tool software V.4.01. CNVs were observed using CNAG viewer (Version 2.0; Takara).

### Association study with p.R4810K (ss179362673)

Association studies were conducted between 161 Japanese cases (41 index cases from Japanese families and unrelated 120 cases) and 384 Japanese controls, between 38 Korean cases (the index case from a Korean family and 37 cases) and 223 Korean controls and between 52 Chinese cases and 100 Chinese controls (Tables S1 and S2). Cases were exclusively diagnosed as moyamoya by RCMJ criteria.

The association was investigated using SNP & Variation Suite V7 (Golden Helix, Inc. in Appendix S1). Allelic, additive, dominant and recessive models were tested. Population-attributable risk (PAR) was defined as PAR  = 100*(K–y)/K, where K is the population prevalence and y is the phenocopy proportion that was estimated from the number of cases with the risk allele [21].

### Screening of *RNF213* polymorphisms in controls

Five *RNF213* variants, p.D4863N, p.E4950D, p.A5021V, p.D5160E and p.E5176G were screened in 757 East Asian controls: 384 Japanese, 223 Korean, and 150 Chinese. Four variants, p.N3962D, p.D4013N, p.R4062Q and p.P4608S, were screened in 384 Caucasian controls: 120 Czech, 164 German, and 100 Caucasian. The screening methods are described in the Text S1 and in Table S5.

### Immortalized cell lines, cell culture and transient transfection

Lymphoblastoid cell lines (LCLs) were isolated and immortalized with Epstein-Barr virus for five unrelated controls (three individuals, the spouse of individual 2 in pedigree 18 and the spouse of individual 12 in pedigree 17) and six cases (individuals 2 and 22 in pedigree 18, individual 11 in pedigree 11 and individuals 12, 121 and 122 in pedigree 17). HeLa cells and human embryonic kidney (HEK) 293 cells were cultured in Dulbecco's modified Eagle's medium (Invitrogen) supplemented with 10% fetal bovine serum. Human umbilical vein endothelial cells (HUVECs) and coronary artery smooth muscle cells (CASMCs) were grown in EBM-2 and SmBM-2 (Lonza), respectively. Expression plasmids were transfected into cells using Lipofectamine LTX (Invitrogen) in accordance with the manufacturer's instructions.

### Cloning of *RNF213* and construction of an expression vector

We cloned *RNF213* and constructed wild-type and various mutants of the *RNF213* expression vector as described in the Text S1. Nucleotide sequence data reported are available in the DDBJ/EMBL/GenBank databases under the accession number AB537889 (NCBI in Appendix S1).

### Detection of splicing products of *RNF213* cDNA or *FLJ35220* cDNA and real-time quantitative PCR

A Human Total RNA Master Panel II (Clontech Inc) and total RNA from the Artery (BioChain) were reverse-transcribed into cDNA using the High Capacity cDNA Reverse Transcription kit (Applied Biosystems). We tested whether *RNF213* cDNA skipped exon 4 of *RNF213* by molecular sizing and direct sequencing of cDNA isolated from the RNA of the above human tissues, HUVECs, and LCLs isolated from the five controls and six cases. The PCR primers were designed to cover exons 3–5 as described in the Text S1. We also tested whether or not *FLJ35220* cDNA skipped exon 11 of *FLJ35220* due to G>A substitution in the intron 11 by molecular sizing of cDNA isolated from LCLs from four cases and three controls.

Quantitative PCR was performed using the THUNDERBIRD SYBR qPCR mix (TOYOBO) and a 7300 Real Time PCR System (Applied Biosystems). The primer sequences for *RNF213* cDNA or *FLJ35220* are described in the Text S1.

### Northern blotting

Total RNA was isolated using a QIAamp RNA blood mini kit (Qiagen Inc.). A human adult normal tissue mRNA northern blot I (Biochain) was probed in accordance with the supplier's recommendations. The two probes [*RNF213*_1 (492 bp) and *RNF213*_2 (591 bp)] have been described in the Text S1. The mRNA levels were determined using an Image Analyzer FLA2000 (Fuji Film).

### Rapid amplification of cDNA ends (RACE)

The 5′ and 3′ ends of the *RNF213* cDNA were determined by RACE using a GeneRacer kit (Invitrogen) according to the manufacturer's protocol. Details can be found in the Text S1.

### Allele-specific mRNA expression assay

Polymorphisms of p.R4810K and a nearby SNP, p.H4557H, in *RNF213* cDNA were used as markers for the SNaPshot assay to measure the expression of the two alleles. Primers for the SNaPshot assay are shown in Table S6.

### Western blotting

Cells were lysed in buffer containing 50 mM Tris-HCl pH 8.0, 1% NP-40 and 150 mM NaCl, or in CelLytic M (Sigma) containing a protease inhibitor cocktail. Samples were subjected to immunoblotting using an anti-RNF213 antibody (MyBioSource), anti-HA antibody (mouse 6E2; Cell Signaling Technology) or anti-Myc antibody (mouse 9E10; Santa Cruz).

### Immunostaining

The methodology used for immunostaining has been described in detail in the Text S1.

### Ubiquitin ligase assay

Plasmids, immunoprecipitation and the self-ubiquitination assay have been described in a previous report [26]. Briefly, cells were lysed in buffer containing 50 mM Tris-HCl pH 8.0, 1% NP-40 and 150 mM NaCl, then centrifuged at 13,000 × *g*. The supernatants were incubated with an anti-HA antibody, and immune complexes were captured using protein G-Sepharose (GE Healthcare). Immunoprecipitates were subjected to immunoblot analysis using an anti-Myc antibody.

### Protein preparation and ATPase assay

The region containing the Walker motif (amino acids 2359–2613) was subcloned into the bacterial expression vector pGEX5X-1. The N-terminal glutathione *S*-transferase (GST)-tagged fragment was first purified with GSH beads (GE Healthcare), then was purified using a gel-filtration column (Superdex 200 prep grade; Amersham Pharmacia) in conjunction with the AKTAexplorer system (GE Healthcare). The ATPase assay was performed as described in Text S1.

### Effects of p.R4810K or p.D4013N on biochemical function of *RNF213*

Nucleotide changes corresponding to p.R4810K and p.D4013N were introduced into *RNF213* using a site-directed mutagenesis kit (Invitrogen). These mutants were transiently expressed in HEK293 cells as described in the Text S1. Cells were then lysed and subjected to the ubiquitin ligase assay as described above, or subjected to subcellular fractionation as described in the Text S1. To generate a RING finger-deleted mutant of *RNF213*, an exogenous *Eco*RI site was fused to the 3′ terminus just before the RING finger domain, then this site was ligated to an endogenous *Eco*RI site located just after the RING finger, enabling the RING finger domain to be skipped from the *RNF213* cDNA.

### Zebrafish model

The Zebrafish experiments were approved by the Osaka University Animal Welfare committee (Osaka University, Japan; approval ID 2197). Embryos and adult fish were raised and maintained under standard laboratory conditions. For *in vivo* experiments, Tg(fli-EGFP)y1 zebrafish [27] were purchased from the Zebrafish International Resource Center. Knockdown of *RNF213-α* and *RNF213-β* expression was achieved by injection of a specific morpholino (MO; Gene Tools) into 1- to 8-cell stage embryos, as described previously [28]. The methods are described in detail in the Text S1.

## Results

### Linkage analysis and identification of the critical region

**Figure 3:**
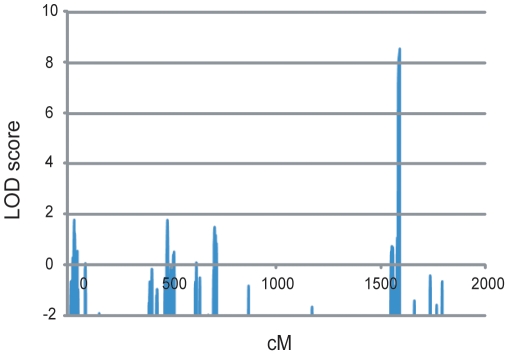
Genome-wide linage analysis uniquely identified a locus on 17q25.3 for the eight families. Genome wide linkage analysis showed a highest LOD score (8.46) at D17S784 in the locus 17q25.3.

Linkage analysis for the eight largest families (Figure 2) revealed a single peak at 17q25.3 with a multipoint LOD score of 8.46 at D17S784 (Figure 3). The largest maximum multipoint LOD score in the 10,000 simulations was 7.25 with a median of 3.56 and a 95% confidence interval between 2.16 and 5.48. Thus, linkage of the eight families to 17q25.3 was highly statistically significant and was unlikely false positive. To evaluate the effect of uncertainty regarding the allele frequency of the disease gene, we conducted a sensitivity analysis by changing the values to 0.000001, 0.001 or 0.01. The sensitivity analysis did not change the maximum multipoint LOD score by more than 1%.

Fine mapping increased the LOD score slightly to 8.52 at D17S784, and revealed several recombination events in the region involving the two flanking markers D17S1806 and rs2280147 (Figure 2). Thus, the core of the locus was a 1.5-Mb region, which harbored 21 genes from *hCG 1776007* to *RPL31P7* (Table S4). The disease haplotype showed complete segregation in the eight families (Figure 2).

### Exome analysis

We generated approximately 154 million reads with an on target rate of 59.1%. Twelve billion bases passed the quality assessment and were aligned to the human reference sequence; >98.1% of the bases mapped to the target, with a mean coverage depth >116.8 in each index case. For coverage on the 1.5-Mb region on 17q25.3, we generated approximately 97000 reads with an on target rate of 52.5%. The mean coverage depth was 48.7 with 288 exons, assuring that all genes were adequately covered.

We applied Ng *et al.* 's filtering algorithm [29], with some modifications to make it less stringent because we anticipated genetic heterogeneity and an uncharacterized gene. Additionally, because a reported discordance of phenotype between identical twins indicated a low penetrance [20], [21], we considered as candidates both rare variants that are specific to moyamoya, and more common variants that are more frequently observed in cases than in controls.

We compared our missense (MS)/nonsense (NS)/splicing site (SS)/insertion or deletion (Indel) variants in the eight index cases against dbSNP 131 (Appendix S1) and the exome database of the five Japanese controls, and removed the reported SNPs and SNPs found in the five Japanese controls (Table 1).

**Table 1:** Numbers of variants identified based on filtering.

		Index Case of the Family								
Stage	Criteria	Ped 2	Ped 10	Ped 14	Ped 15	Ped 17	Ped 18	Ped 19	Ped 20	Any eight
1st Stage	MS/NS/SS/Indel	6638	6741	6512	6668	6561	6361	6822	6507	1038
	Not in dbSNP131	983	1036	963	966	953	1064	1019	934	75
2nd Stage	Not in the five Japanese controls*	583	590	565	611	594	534	624	605	2 : p.N321S in PCMTD1 and p.R4810K in RNF213
	No of genes	377	379	364	372	375	354	380	385	2
	In the 17q25.3	1	1	1	1	3	3	1	2	1
	Genes No of genes	RNF2131	RNF2131	RNF2131	RNF2131	RNF213GAAENPP73	RNF213ENPP7SLC26A113	RNF2131	RNF213SLC26A112	RNF2131

Rows show the effect of excluding from consideration of variants found in dbSNP131 and the five Japanese controls. Columns show MS/NS/SS/Indel variants that were observed in each affected individual. The column 10 provided observation that shared by all affected index cases.

*Exome analysis were conducted using the same platform and same experimental conditions for five Japanese controls.

The mean numbers of MS/NS/SS/Indel per cases were 6601 from the genome-wide analysis. We next examined the effects on the size of the candidate gene list when analyzing the exomes of the eight index cases in various combinations and examined the potential consequences of genetic heterogeneity, such that only a subset of the exomes of index cases was required to contain new variants in a given gene for it to be considered as a candidate gene (Table 1 and S7). In the second stage of the filtering in which we assumed that any seven of the eight index cases shared the causative gene (Table S7), the numbers of the candidate genes were decreased to two. By filtering two variants in the two genes emerged as candidates (Table 1 and Table S7). These two variants were p.N321S in *PCMTD1* (Chr8: NM_052937) and p.R4810K in *RNF213* (Chr17: NM_020914.4). *PCMTD1* encodes the protein-L-isoaspartate O-methyltransferase domain-containing protein 1(Genecard in Appendix S1) ; *RNF213* encodes ring finger protein 213. We thus considered both *PCMTD1* and *RNF213* as candidate genes.

### Confirmation of the exome data by direct sequencing and segregation analysis

In the next step, we conducted sequencing to confirm p.N321S in *PCMTD1* and sequenced the entire exons of *RNF213* with an ORF (NM_020914.4) in 42 index cases by the Sanger method (Table S3). We could not confirm p.N321S in *PCMTD1* in any of the eight index cases by the Sanger method. Thus we discarded *PCMTD1*. In contrast, we could confirm p.R4810K by the Sanger method in 42 index cases but could not find any other unregistered polymorphism in *RNF213*. We genotyped other family members in the 42 families. As shown in Figure S1, p.R4810K was completely segregated in 42 families: all the affected members by RCMJ criteria had p.R4810K, although some carriers were not affected with moyamoya disease, suggesting low penetrance.

### Deep sequencing around *RNF213*

To avoid a possible pitfall of exome for searching variants in introns or intergenic regions, we sequenced the entire 260-kb genomic region (UCSC Genome Browser in Appendix S1) around *RNF213* from the 5′ end of *SLC26A11* to the 5′ end of *NPTX1* in the index case of pedigree 11 using BAC clones or direct sequencing. Sequencing the 2.7-kb intron 15 of *RNF213* failed because of the presence of repeats. Southern blotting of the eight index cases and a control (the spouse of individual 2 in pedigree 18) confirmed the absence of insertions or deletions in this intron (data not shown). However, sequencing of the 260-kb genomic region revealed three additional unregistered variants in noncoding regions: ss179362671 (T>C) in intron 15 of *RNF213*, ss179362674 (G>A) in intron 11 of *FLJ35220* and ss179362675 (C>T) at the 3′ end of *NPTX1*, 1868 bp downstream from the 3′UTR.

### Exonic rare variants of *RNF213* in Japanese controls

We then sequenced all the exons of *RNF 213* in ten controls and conducted deep sequencing of *RNF213* for a Japanese control. These results and five controls for exome analysis are shown in Table S8. Additionally, 38 chromosomes inherited from parents in 38 index cases, which do not carry p.R4810K, can be identified to have been transmitted from control parents. Pooling these data, an observed sample size of control chromosomes accounts for 70 (five controls by exome, 10 controls by direct sequencing, one by deep sequencing, and 38 chromosomes inherited from unaffected parents of 38 index cases). Given that the statistical power should be greater than 80%, rare exonic variants with minor allele frequencies of as low as 2% could be detected in the sample size of 70. However, we could not find any unregistered rare variants on control chromosomes.

### Possible masked variants in the linkage disequilibrium (LD) region with p.R4810K (ss179362673) and possible CNVs in 17q25.3

Although p.R4810K seems to be a strong candidate for causative variants of moyamoya disease, there also seem to be other possibilities. One of these is the presence of masked variants in the promoters, or intronic or intergenic regions of a bona fide causal gene, which are in strong LD with p.R4810K.

**Figure 4:**
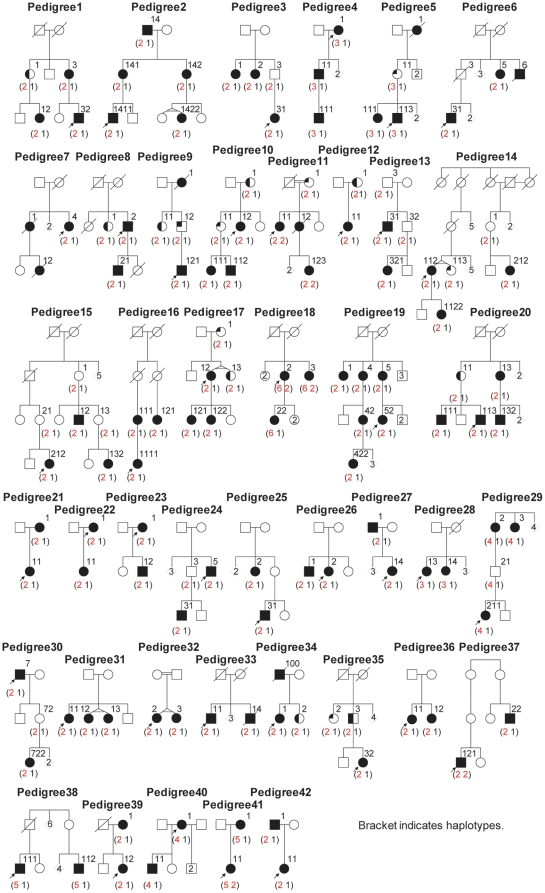
Transmission patterns of the haplotypes in 42 families. The numbers in the parentheses indicate the haplotypes transmitted in each family. Haplotype 1 symbolizes non-risk haplotypes collectively. Therefore it comprises various haplotypes. Haplotypes can be seen in Figure 5. Several family members that were genotyped were omitted in the pedigree chart for clarity. Those members and their haplotypes were; two siblings (1,1) for pedigree 7; father (1,1) for pedigree 21; father (1,1) for pedigree 22; father (1, 1) and a sibling (1,1) for pedigree 23; father (1,1), mother (2,1) and a sibling (1,1) for pedigree 26; three siblings (1,1), (1,1) and (1,1) for pedigree 27; father (1,1), three siblings (1,1) (1,1) and (1,1) for pedigree 28; father (1,1), mother (2,1) and a child of 2 (1,1) for pedigree 32; three siblings (1,1), (1,1), and (1,1) for pedigree 33; mother (1,1) for pedigree 34; father (1,1) and mother (2,1) for pedigree 36; a sibling (1,1) for pedigree 39; three siblings (1,1), (4,1) and (1,1) for pedigree 40; father (2,1) for pedigree 41; mother (1,1) for pedigree 42.

**Figure 5:**
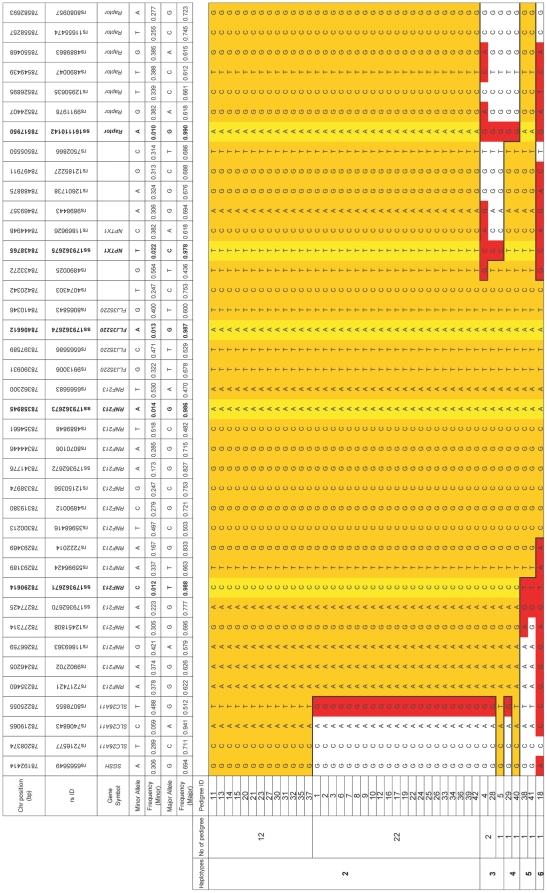
Risk haplotypes transmitted in 42 families. The orange regions represent the haplotype of the index case of pedigree 11. The yellow regions indicate rare variants. The red and white regions represent flanking SNPs and SNPs outside of the founder haplotype, respectively. The minimum founder haplotype fell in a a region in a span of 130 kb covering *RNF213* and *FLJ35220*. The physical positions were referred from Build 37.1.

We determined the minor allele frequencies for five unregistered variants found in this study and a previous study [21] in Japanese controls. The frequencies were 0.012 (p1 = 9/768) for ss179362671, 0.014 (p2 = 11/768) for ss179362673, 0.013 (p3 = 10/768) for ss179362674, 0.022 (p4 = 17/768) for ss179362675 and 0.010 (p5 = 8/768) for ss161110142 in 384 Japanese controls, indicating that they are all rare variants. We then genotyped the 42 families. As shown in Figure 4, these four rare variants were transmitted en bloc with p.R4810K. They comprised five subhaplotypes (Figure 5); and p.R4810K was not transmitted alone in any of the families.

Haplotype 2, the most prevalent haplotype, was found in 34 of the 42 families. Because all five variants are rare, it is unlikely that haplotype 2 has been formed by chance (p = 2^5^×p1×p2×p3×p4×p5 = 1.5×10^−8^). Alternatively, a more rational explanation is to assume that haplotype 2 is the founder haplotype. Along this line, haplotypes 3–6 may be explained by historical recombination events. Given historical recombinations, the number of masked variants is very likely to be one and it should be located in the core LD region between ss179362671 and ss179362675 (Figure 5).

Further confirmation of the sequence homogeneities was evaluated by genotyping of 34 additional markers. Transmissions of the 39 SNPs covering *SGSH* to *Raptor* were investigated in the 42 families. The five haplotypes were further classified into subtypes (Figure 5). The typing results confirmed the sequence homogeneity in the core LD region (Figure 5). The genotyping of the SNPs in the *RNF213* regions were completely in accord with the sequencing results in the 42 index cases. Thus, we concluded that the core LD region has been very likely derived from the founder haplotype.

Although we sequenced the coding and non-coding regions in the index case of pedigree 11, we did not detect any unregistered rare variants other than the five rare variants, supporting that p.R4810K or G>A substitution in the intron 11 of *FLJ35220* is a susceptibility variant. In terms of G>A substitution in the intron 11 of *FLJ35220*, we have tested two possibilities, namely that the substitution may induce aberrant splicing or change gene expression levels. Either possibility was discarded (Figure S3). We still tested another possibility. It can sometimes be difficult to detect CNVs by direct sequencing. Therefore, we evaluated the CNVs using a high-density microarray, with a theoretical sensitivity estimated to be 41 kb [30]. However, we did not detect any CNVs in the 17q25.3 region in the three index cases of pedigrees 5, 11 and 18 and a control 2 in pedigree 18 (Figure S4).

Although we cannot theoretically discard the very rare possibility of multiple masked variants having strong LD with p.R4180K by various LD patterns specific to the individual families, these data collectively indicate that it is not a surrogate marker of possible masked variants of another gene. Therefore, *RNF213* is very likely to be a bona fide susceptibility gene for moyamoya disease.

### Association of p.R4810K with moyamoya disease in East Asian patients

We next tested the association of *RNF213* p.R4810K with moyamoya disease in East Asian populations. Unrelated cases and index cases were exclusively limited to those who met the diagnostic criteria for definitive moyamoya disease [22]. As shown in Table 2, ss179362673 (p.R4810K in *RNF213*) was significantly associated with moyamoya disease, with a maximum odds ratio (OR) of 338.9 (*P* = 10^−100^). The association was perfectly replicated in the Korean (OR = 135.6, *P* = 10^−26^) and Chinese (OR = 14.7, *P* = 10^−4^) populations (Table 2). Stratification by family histories of moyamoya disease did not substantially change the association of p.R4810K with moyamoya disease (Table S9).

**Table 2:** Association of ss179362673 with moyamoya disease.

Ethnicity	HWE P Cases	HWE P Controls	DD Cases	DD Controls	Dd Cases	Dd Controls	dd Cases	dd Controls	Sample Size
Japanese	0.00	0.00	10	1	135	9	16	374	545
Korean	0.00	0.84	0	0	30	6	8	217	261
Chinese	0.81	0.92	1	0	11	2	40	98	152
Total	0.00	0.01	11	1	176	17	64	689	958
Model	Ethnicity	Chi-Squared (-log_10_P)	OR (Minor Allele)	Lower CI	Upper CI	Minor Allele Frequency (Cases)	Minor Allele Frequency (Controls)		
Allelic	Japanese	84.63	63.87	33.88	120.42	0.48	0.01		
	Korean	33.11	47.83	18.91	120.93	0.40	0.01		
	Chinese	4.95	14.14	3.13	63.98	0.13	0.01		
	Total	117.73	47.82	29.39	77.81	0.39	0.01		
Additive	Japanese	100.20	244.58	113.98	525.32				
	Korean	26.12	135.63	43.03	427.52				
	Chinese	4.64	13.69	2.86	65.56				
	Total	117.66	97.11	56.15	168.01				
Dominant	Japanese	99.98	338.94	147.82	777.44				
	Korean	26.12	135.63	43.03	427.52				
	Chinese	4.58	14.70	3.05	70.81				
	Total	119.18	111.84	64.01	195.39				
Recessive	Japanese	4.84	25.36	3.09	208.30				
	Korean	0.92	0.36	0.08	1.61				
	Chinese	0.10	0.88	0.33	2.36				
	Total	5.91	32.36	3.99	262.70				

OR: odds ratio; CI: 95% Confidence Interval; HWE: Hardy Weinberg Equilibrium; DD: minor allele homozygote; Dd: heterozygote; dd: major allele homozygote; SE: Standard error.

### Variant searching in non-p.R4810K East Asian cases and in Caucasian cases

Although p.R4810K was identified in three ethnic populations, the population attributable risks in the Japanese (145/161; 90%) and Korean (30/38; 79%) populations were larger than that in the Chinese population (12/52; 23%). Additionally, we showed by genotyping that the p.R4810K variant was not present among Caucasian cases or controls. These pieces of evidence strongly suggest genetic heterogeneity. We thus sequenced *RNF213* to identify other variants in non-p.R4810K East Asian cases and Caucasian cases.

By direct sequencing, five distinct *RNF213* variants, i.e., p.D4863N, p.E4950D, p.A5021V, p.D5160E and p.E5176G, were identified in seven out of 64 East Asian cases (40 Chinese, 16 Japanese and 8 Korean) (Table 3, Figure S5). None of these variants was found among 757 East Asian controls (Table 3). In Caucasians, four distinct variants, i.e., p.N3962D, p.D4013N, p.R4062Q and p.P4608S, were identified in 4 out of 50 (8%) cases (Table 3): in the index case of the Caucasian pedigree (Figure S2A) and 3 out of 49 other cases (Figure S5). In the Czech family, the p.D4013N (G>A) variant was transmitted to the three affected children and segregated perfectly with moyamoya disease (Figure S2). None of these variants was found among 384 Caucasian controls (Table 3).

**Table 3:** Summary of the variants in *RNF213.*

	Variant	78358945	78360097	78360619	78363034	78367154	78367201	78341560	78341825	78343331	78355371	Total
		G>A	G>A	G>C	C>T	C>G	A>G	A>G	G>A	G>A	C>T	
	Effect*	p.R4810K	p.D4863N	p.E4950D	p.A5021V	p.D5160E	p.E5176G	p.N3962D	p.D4013N	p.R4062Q	p.P4608S	
Japanese	Case (n = 161)	145 (10)	0	0	0	0	0	0	0	0	0	145
	MAF	48.1	0	0	0	0	0	0	0	0	0	48.1
	Control (n = 384)	10 (1)	0	0	0	0	0	ND	ND	ND	ND	10
	MAF	1.4	0	0	0	0	0	ND	ND	ND	ND	1.4
Korean	Case (n = 38)	30 (0)	0	0	0	0	0	0	0	0	0	30
	MAF	39.5	0	0	0	0	0	0	0	0	0	39.5
	Control (n = 223)	6 (0)	0	0	0	0	0	ND	ND	ND	ND	6
	MAF	1.3	0	0	0	0	0	ND	ND	ND	ND	1.3
Chinese	Case (n = 52)	12 (1)	1	2	2	1	1	0	0	0	0	19
	MAF	12.5	1	1.9	1.9	1	1	0	0	0	0	19.3
	Control (n = 150)	2 (0)	0	0	0	0	0	ND	ND	ND	ND	2
	MAF	0.7	0	0	0	0	0	ND	ND	ND	ND	0.7
Total in East Asian	Case (n = 251)	187	1	2	2	1	1	0	0	0	0	194
	%	74.5	0.4	0.8	0.8	0.4	0.4	0	0	0	0	77.3
	Control (n = 757)	18	0	0	0	0	0	ND	ND	ND	ND	18
	%	2.4	0.0	0.0	0.0	0.0	0.0	ND	ND	ND	ND	2.4
Czech	Case (n = 8)	0	0	0	0	0	0	0	1	0	0	1
	MAF	0	0	0	0	0	0	0	6.3	0	0	6.3
	Control (n = 120)	0	ND	ND	ND	ND	ND	0	0	0	0	0
	MAF	0	ND	ND	ND	ND	ND	0	0	0	0	0
German	Case (n = 42)	0	0	0	0	0	0	1	0	1	1	3
	MAF	0	0	0	0	0	0	1.2	0	1.2	1.2	3.6
	Control (n = 164)	0	ND	ND	ND	ND	ND	0	0	0	0	0
	MAF	0	ND	ND	ND	ND	ND	0	0	0	0	0
Caucasian	Control (n = 100)	0	ND	ND	ND	ND	ND	0	0	0	0	0
	MAF	0	ND	ND	ND	ND	ND	0	0	0	0	0
Total in Caucasian	Case (n = 50)	0	0	0	0	0	0	1	1	1	1	4
	%	0	0	0	0	0	0	2	2	2	2	8
	Control (n = 384)	0	ND	ND	ND	ND	ND	0	0	0	0	0
	%	0	ND	ND	ND	ND	ND	0	0	0	0	0

*Based on AB537889.

ND: Not determined.

() :Number of homozygotes.

MAF: Minor allele frequency.

**Figure 6:**
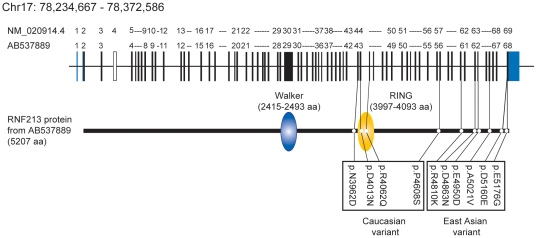
Genomic structure, domains of *RNF213* and variants. Genomic structure was based on DDBJ/EMBL-Bank/GenBank accession number AB537889. Domain structure was obtained by GeneCards.

In total, our extensive sequencing analysis identified 10 variants in *RNF213* (Table 3). These variants were all located in the 3′ half of the gene (Figure 6). We concluded that *RNF213* is a susceptibility gene for moyamoya disease.

### Full-length cDNA cloning of *RNF213*

Because of its large size, *RNF213* was first cloned as five separate fragments, which were then connected using internal restriction enzyme sites, as indicated in Figure S6. The 5′ and 3′ ends of cDNA from the LCL of the index case of pedigree 11 were determined by RACE, which defined the transcriptional start site and the 3′ end of the *RNF213* gene as at nucleotides 78,234,667 and 78,370,086, respectively (UCSC Genome Browser in Appendix S1). Thus, the full length *RNF213* was found to have a 15624-bp ORF and 5431-bp 5′ and 3′UTR. The whole cDNA of *RNF213* [AB537889] is similar in size to the cDNA of *RNF213* [NM_020914.4], but there are differences: it lacks exon 4 and has a 3′UTR 2500 bp longer than that of NM_020914.4 (NCBI in Appendix S1).

**Figure 7:**
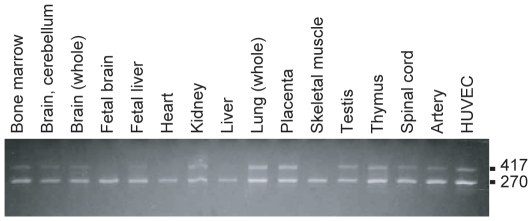
Two isoforms of alternative splicing variants of *RNF213.* We have tested whether exon 4 is read through or not in cDNA isolated from various human tissues and HUVECs. Representative results of human tissue RNAs and HUVEC are shown. A short isoform, which skips the exon 4, has an expected size of 270 bp (AB537889) and a long, which reads exon 4, has an expected size of 417 bp (NM_020914.4).

Two splicing variants of *RNF213* were detected in cDNAs isolated from bone marrow, cerebellum, whole brain, fetal brain, fetal liver, heart, kidney, liver, lung, placenta, skeletal muscle, testis, thymus, spinal cord, artery and HUVECs (Figure 7). The same splicing variants were also detected in LCLs isolated from the five controls and the six cases (data not shown). These two isoforms correspond to the short isoform (270 bp) which skips exon 4 (DDJB, AB537889) and the long isoform (417 bp) which reads exon 4 (NM_020914.4) (Figure 7). The short isoform is the major splicing variant and detected in all tissues examined, but the long isoform is a minor and not found in several tissues.

*In silico* analysis revealed two well-known domains, a RING finger domain and a Walker motif, which are reported to exhibit ubiquitin ligase activity and ATPase activity, respectively (Figure 6) [31],[32] (Genecard in Appendix S1).

### Expression profile of *RNF213* and functional characterization

**Figure 8:**
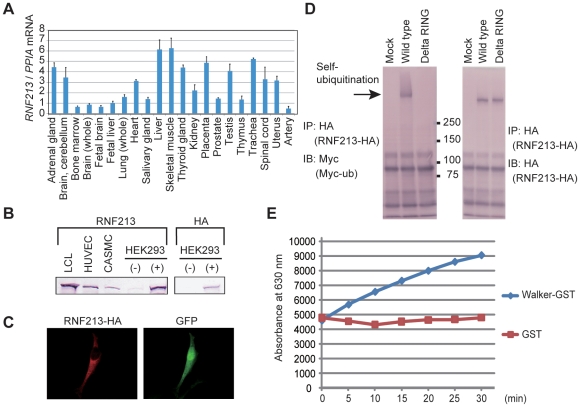
Characterization of the *RNF213* gene and protein. (**A**) *RNF213* mRNA expression. Total RNA from the indicated human tissues was reverse-transcribed to cDNA, and real-time quantitative PCR was performed. (**B**) RNF213 protein expression. LCL, HUVEC, and CASMC were lysed and immunoblotted using an anti-RNF213 antibody. HEK293 cells transiently expressing RNF213-HA (+) or control cells (−) were immunoblotted using anti-RNF213 and anti-HA antibodies. (**C**) Subcellular localization of RNF213. HeLa cells transiently expressing RNF213-HA were stained with an anti-HA antibody. (**D**) Self-ubiquitination of RNF213. HEK293 cells transiently expressing RNF213-HA and Myc-ubiquitin (Myc-ub) were lysed and subjected to immunoprecipitation (IP) using an anti-HA antibody, followed by immunoblotting (IB) using an anti-Myc antibody. As a control, immunoblotting was also performed with an anti-HA antibody. (**E**) ATPase activity of RNF213. Free phosphate released from ATP by the ATPase activity of a recombinant RNF213 fragment (a.a. 2359–2613) tagged with GST was measured using the Malachite Green method. a.a., amino acid. IP, immunoprecipitation. IB, immunoblot.

*RNF213* mRNA is expressed in various human tissues (Figure 8A). Northern blotting revealed the expression of full-length *RNF213* in several human tissues (Figure S7A), cultured cell lines and LCLs (Figure S7B). We also detected the expression of the 591-kDa endogenous RNF213 protein in LCLs, HUVECs, CASMCs and HEK293 cells by western blotting using a RNF213-specific antibody. The western blot band size was consistent with that of overexpressed HA-tagged RNF213 (Figure 8B).

Transient transfection of HA-tagged *RNF213* into HeLa cells resulted in expression of the protein throughout the cytosol (Figure 8C), with partial association with the intracellular membrane and cytoskeleton (Figure S8B). The E3 activity of the RING finger domain was confirmed by self-ubiquitination after transfecting full-length *RNF213* cDNA into HEK293 cells (Figure 8D). ATPase activity was detected *in vitro* by free phosphate releasing analysis using a recombinant RNF213 fragment including the Walker motif (Figure 8E). Thus, *RNF213* is a unique protein, and we report here for the first time that it is a single protein that possesses both ubiquitin ligase activity and ATPase activity.

### Homology search

A homology search revealed conservation of the arginine residue at position 4810 of *RNF213* in mammals (Table 4) (BLAST in Appendix S1). Three variants: p.R4062Q, which is located in the RING finger domain, p.R4810K and p.E5176G, were conserved among most species (Table 4). Variants p.N3962D, p.P4608S, p.D4863N, p.E4950D, p.A5021V, and p.D5160E were outside the RING finger domain and not conserved across species (Table 4). It should be noted that for five variants (p.N3962D, p.D4013N, p.E4950D, p.A5021V, and p.D5160E), the variant allele was present at the equivalent position in at least one of the species examined.

**Table 4:** Homology of *RNF213.*

East Asian variant									
Species		Gene	Accession number	R4810K	D4863N	E4950D	A5021V	D5160E	E5176G
*Homo sapiens*	Human	*RNF213*		4803 QVEYSSI**R**GFLSKHS 4817	4856 CSTDLDL**D**TEFEILL 4870	4943 EGRETVQ**E**FDLEKIQ 4957	5014 QLQSYSD**A**CEVLSVV 5028	5153 RPQWSLR**D**TLVSYMQ 5167	5169 KESEILP**E**MASQFPE 5183
*Homo sapiens*	Human	*RNF213*	NP_065965.4	4852 QVEYSSI**R**GFLSKHS 4866	4905 CSTDLDL**D**TEFEILL 4919	4992 EGRETVQ**E**FDLEKIQ 5006	5063 QLQSYSD**A**CEVLSVV 5077	5202 RPQWSLR**D**TLVSYMQ 5216	5218 KESEILP**E**MASQFPE 5232
*Pan troglodytes*	Chimpanzee	*RNF213*	XP_511726.2	3745 QVEYSSI**R**GFLSKHS 3759	3798 CSTDLDL**D**TEFEILL 3812	3885 EGRETVQ**E**FDLEKIQ 3899	3956 QLQSYSD**A**CEVLSVV 3970	4095 RPQWSLR**D**TLVSYMQ 4109	4111 KESEILP**E**MASQFPE 4125
*Mus musculus*	Mouse	mCG142721, isoform CRA_a	EDL34702.1	4828 VEYSSI**R**GFIHSHS 4841	4880 CCSDLDL**D**AEFEVIL 4894	4967 QGGETSQ**E**FDLEKIQ 4981	5038 QLQSYSD**A**CEALSII 5052	5175 NPNWSLK**D**TLVSYME 5189	5191 KDSDILS**E**VESQFPE 5205
*Rattus norvegicus*	Rat	*RNF213*	XP_001081768.2	4828 EVEFSSI**R**SFIHSHHS 4843	4881 CRSDLDL**D**AKFEVIL 4895	4968 QGGETSQ**E**FDLEKIQ 4982	5039 QLQSYSD**A**CEALSIV 5053	5176 NPSWSLK**D**TLVSYME 5190	5192 KDSDVLT**E**VESQFPD 5206
*Bos taurus*	Cow	similar to *mCG142721*, partial	XP_590465.5	4681 EVEYKSI**R**SFISSH 4694	4734 CSSDLDL**D**TDLEVIL 4748	4821 QGGETLQ**E**FDLEKIQ 4835	4892 QLQSYSD**A**CEALSAT 4906	5029 NPEWSLR**D**TLVSYME 5043	5045 TDSEIPP**E**MESQFPE 5059
*Canis familiaris*	Dog	*RNF213*	XP_540474.2	2986 EADYQSI**R**SFISSHQ 3000	3039 CSADLDM**D**TNFEVIL 3053	3126 QGKETLQ**E**FDLEKIQ 3140	3197 QLPSYSD*G*CKALSVI 3211	3334 RPEWSLR**D**TLVSYME 3348	3350 KDSEIPP**E**LEYQFPE 3364
*Monodelphis domestica*	Kangaroo	hypothetical protein LOC100030710	XP_001380151.1	4718 VEYNTI**R**GFL 4727	4770 CDADLSL*E*NEFEILL 4784	4857 KGRETLQ*Q*FDLEKIQ 4871	4928 QLQSYSD**A**CEALSVT 4942	5066 NPKWSLK*E*TLVSYME 5080	5082 KESEIPP**E**VEYQFPE 5096
*Taeniopygia guttata*	Zebra finch	*RNF213*	XP_002192487.1	4254 EIKHCSI**R**EFLREPH 4268	4307 CDAELSL*E*SRLEVLL 4321	4394 KGGETLQ*D*FDLERIQ 4408	4465 ELQSYSD*V*CDALSLT 4479	4603 KSTWSLK*E*SLLPYLY 4617	4618 KDSELTL**E**LEDTFPD 4632
*Danio rerio*	Zebrafish	*RNF213*	XP_001921030.2	3888 DLTYKTI**R**EFLQDQK 3902	3941 TEKDLGL**D**ADLQVLL 3955	4028 CGQETLL**E**YDLPKIQ 4042	4099 ELQSYSD*V*CEALSTV 4113	4237 RPDWRLK*H*TVVSYME 4251	4253 KDLDVPP**E**VEEFFPK 4267
*Takifugu rubripes*	Fugu	*C17of27*	AAL32171.1	3881 AVQTGTI**R**EFLNTQN 3895	3934 CQSDMDH*S*SDFSFLL 3948	4021 RGQESLL**E**YDLAKLQ 4035	4092 ELQSYSE*V*CEALSTL 4106	4229 KPEWSLA*V*TLFSYME 4243	4245 KDLDVSP**E**M-EEFPE 4259
*Tetraodon nigroviridis*	Spotted green pufferfish	unnamed protein product	CAG00202.1	892 ELS-ATIEEFLNTQN 905	944 CQSDMDL*S*SDFRVLL 958	1031 KGQETLP**E**YDLAKIQ 1045	1102 ELQSYSE*V*CEALSTL 1116	1242 TGASRTR*S*PLTWSAK 1256	1242 TGASRTR*S*PLTWSAK 1256
Caucasian variant									
Species		Gene	Accession number	N3962D	D4013N	R4062Q	P4608S		
*Homo sapiens*	Human	*RNF213*		3955 LDKCLRE**N**SDVKTHG 3969	4006 DPVCLPC**D**HVHCLRC 4020	4055 IEKHARF**R**QMCNSFF 4069	4601 LINIIKP**P**VRDPKGF 4615		
*Homo sapiens*	Human	*RNF213*	NP_065965.4	4004 LDKCLRE**N**SDVKTHG 4018	4055 DPVCLPC**D**HVHCLRC 4069	4104 IEKHARF**R**QMCNSFF 4118	4650 LINIIKP**P**VRDPKGF 4664		
*Pan troglodytes*	Chimpanzee	*RNF213*	XP_511726.2	2901 LDKCLRE**N**SDVKTHG 2915	2952 DPVCLPC**D**HVHCLRC 2966	3001 IEKHARF**R**QMCNSFF 3015	3543 LINIIKP**P**ARDPKGF 3557		
*Mus musculus*	Mouse	mCG142721, isoform CRA_a	EDL34702.1	3959 LDKCLEE*D*SNLKT 3971	4010 DPVCLPC**D**HVYCLRC 4024	4059 IEKHAQF**R**HMCNSFF 4073	4629 LTVIIKP*W*VQDPQGF 4643		
*Rattus norvegicus*	Rat	*RNF213*	XP_001081768.2	3959 LDKCLEE*D*SNLKT 3971	4010 DPVCLPC**D**HVYCLPC 4024	4059 IEKHAQF**R**HMCNSFF 4073	4630 LMNIIKP**P**VQDPQGF 4644		
*Bos taurus*	Cow	similar to *mCG142721*, partial	XP_590465.5	3837 LNKCLLE*D*SDTKTH 3850	3888 DPVCLPC**D**HIFCLRC 3902	3937 IEKHARF**R**QMCNSFF 3951	4483 LMNIIKP**P**VSDPKRF 4497		
*Canis familiaris*	Dog	*RNF213*	XP_540474.2	2142 LNKYLQD*D*SDIKTYRP 2157	2193 EPVSLPC*G*HVFCLRC 2207	2242 IRKHACL**R**QMCNSFF 2256	2784 VMNIIKP**P**VRDPSSF 2798		
*Monodelphis domestica*	Kangaroo	hypothetical protein LOC100030710	XP_001380151.1	3868 LGKCLQD**N**SDIKTH 3881	3919 EPVCLPC**D**HVYCQKC 3933	3968 IAKHIQF**R**QMCNSFF 3982	4515 LMKIIKP**P**VRDPESF 4529		
*Taeniopygia guttata*	Zebra finch	*RNF213*	XP_002192487.1	3441 LAKCFQL*D*SDMKSHP 3455	3492 DPICLPC*N*HVFCHKC 3506	3540 IAKKALF**R**QRCNNFF 3554	4053 LGSMIKP*T*VKNVVSF 4067		
*Danio rerio*	Zebrafish	*RNF213*	XP_001921030.2	3046 LAQVLEQ*D*SNLKKKK 3060	3097 DPLSLPC**D**HIYCLTC 3111	3146 ISQNASF**R**MRCNAFF 3160	3687 IMQIIKP*A*VVHPDAF 3701		
*Takifugu rubripes*	Fugu	*C17of27*	AAL32171.1	3040 LGQILEK**N**SDLKTYE 3054	3091 DPLCLPC**D**HIYCQAC 3105	3140 VNQHARF**R**KQCNAFF 3154	3681 GSAIIKP*V*VHDPGAF 3695		
*Tetraodon nigroviridis*	Spotted green pufferfish	unnamed protein product	CAG00202.1	65 LGQILEKSSDLKSHQ 79	116 EPLSLPC**D**HIYCLGC 130	165 VNQHAQF**R**KRCNAFF 179	668 VSAIIKP*Q*VSDPGVF 682		

Orthologue genes were searched by BLAST.

### Biochemical effects of p.R4810K and p.D4013N

We tried to characterize the p.R4810K and p.D4013N allele proteins of *RNF213 in vitro.* We first investigated stability of the p.R4810K variant. The wild-type or p.R4810K variant of RNF213-HA was transiently expressed in HEK293 cells. Cells were lysed and subjected to immunoblotting with an anti-HA antibody. As shown, the molecular stability in cells was not altered (Figure S8A). Next we investigated subcellular localization of the p.R4810K variant of *RNF213*. HEK293 cells transiently expressing the wild-type or p.R4810K variant of RNF213-HA were fractionated into cytosol, membrane/organelle, nucleus, and cytoskeleton using different lysis reagents (ProteoExtract kit; Calbiochem). However, we could not detect alteration of intracellular distribution of p.R4810K (Figure S8B). Next we examined whether self-ubiquitination of the p.R4810K variant or p.D4013N mutant of *RNF213* was altered. HEK293 cells transiently expressing the wild-type or p.R4810K variant or p.D4013N mutant of RNF213-HA and Myc-ubiquitin were lysed and subjected to immunoprecipitation using an anti-HA antibody, followed by immunoblotting using an anti-Myc antibody. Neither of the two variants was found to alter ubiquitin activities (Figure S8C and S8D).

### Allele-specific mRNA expression of *RNF213*

To determine whether *RNF213* mRNA from the p.R4810K (G>A) allele is specifically expressed in moyamoya patients, a SNaPshot assay was performed (Figure S9). The allele specific ratios of p.R4810K were between 1.03 and 1.19, suggesting that there is no allele-specific gene expression in moyamoya patients.

### *RNF213* orthologues in zebrafish

We could not prove detrimental effects of the variants. Therefore we aimed to obtain further insight into the physiological function of *RNF213* by suppressing its gene expression in zebrafish. In zebrafish, two *RNF213* genes, *RNF213-α* and *RNF213-β* are located on different chromosomes as a result of whole-genome duplication. The predicted amino acid sequences indicate that both *RNF213* genes are human *RNF213* orthologues (UCSC Genome Browser in Appendix S1), with nearly perfect conservation in the Walker and RING finger motifs. These genes are highly related to one another at the amino acid level, with *RNF213-α* and *RNF213-β* sharing similar exon structures. However, comparisons of exon-intron boundaries reveal low conservation in these *RNF213* MO target sequences. Therefore we could design a MO that specifically knocks down each *RNF213* gene. Expression analysis by RT-PCR indicated that *RNF213-α* was expressed to a greater extent than *RNF213-β* (Figure S10). *In situ* hybridization analysis was not successful, probably because of weak or scattered expression of each *RNF213*.

To knock down these *RNF213* orthologues, MO nucleotides were designed to specifically target splice sites (sp-MO). Two pairs of *RNF213-α* sp-MO and a *RNF213-β* sp-MO successfully interfered with the splicing of *RNF213* transcripts (Figure S10). To assay vascular development, we used the Tg(fli-EGFP)y1 line, in which endothelial cells are marked by EGFP expression. Injection of both sp-MO pairs against *RNF213-α* (pair 1: *RNF213-α-*MO1-A and MO1-D, pair 2: *RNF213-α-*MO2-A and MO2-D) elicited very similar abnormal vascular development (Figure S11). About three quarters of embryos injected with pairs of *RNF213-α* morpholinos (2.5 ng each) presented with similar vascular anomalies by 72 hpf (73% of pair 1, n = 177, and 76% of pair 2, n = 43). By contrast, injection of a sp-MO against *RNF213-β* resulted in normal vascular development, presumably because little *RNF213-β* is expressed *in vivo* (Figure S11).

### *RNF213* knock-down zebrafish

**Figure 9:**
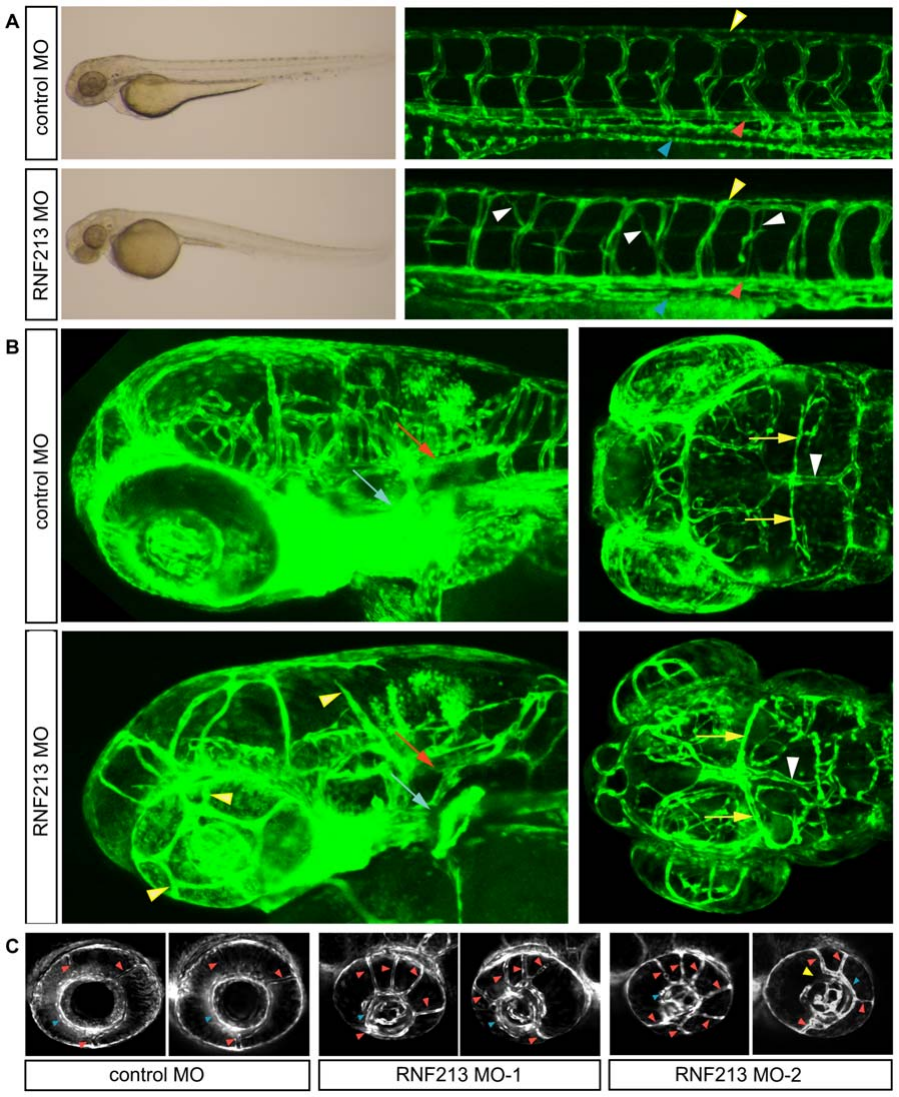
Depletion of *RNF213* causes abnormal vessel sprouting in zebrafish. Tg(fli-EGFP)y1 embryos at 72 h post-fertilization. (**A**) Brightfield image of whole embryos (left) and confocal images of tail vessels (right) of embryos injected with a control (scrambled) or *RNF213* morpholino (MO). Lateral views, dorsal uppermost, anterior to the left. In *RNF213* morphants, abnormal intersegmental vessel sprouting was observed (white arrowheads). Trunk vessels, including the dorsal aorta (red arrowheads), posterior cardinal vein (blue arrowheads) and dorsal longitudinal anastomotic vessels (yellow arrowheads) developed almost normally. (**B**) Confocal images of intracranial vessels. (Left) Lateral views 20 degrees toward the top; dorsal uppermost, anterior to the left. (Right) Dorsal views, head to the left. The trunk artery, including the basilar artery (red arrows), lateral dorsal aorta (blue arrows), mesencephalic artery (yellow arrows) and dorsal longitudinal vein (white arrowheads) developed almost normally in controls and *RNF213* morphants. *RNF213* morphants showed abnormal sprouting vessels (yellow arrowheads) and irregular vessel diameter (white arrowheads). (**C**) Cross-sectional view at the middle of the lens level. In a control morphant, the three branches of the nasal ciliary artery (red arrowheads) drain into the inner optic circle (IOC; blue arrowheads). In two different *RNF213* morphants, multiple aberrant vessels drain into the IOC (red arrowheads), and occasionally part of the IOC was missing (yellow arrowhead).

In bright-field images, *RNF213* morphants showed a slight reduction in body size, a small eye, and a wavy trunk compared to the control (Figure 9A, left). Formation of the axial trunk vessels, the dorsal aorta and posterior cardinal vein proceeded almost normally, indicating that arteriovenous specification was not affected. Intersegmental vessel sprouts emerged from the dorsal aorta at regular positions; however, the elongating sprouts did not track closely to intersegmental boundaries and sometimes reached dorsal longitudinal anastomotic vessels at the next intersegmental boundary, although somite boundaries appeared morphologically normal (Figure 9A, right). These phenotypes were not caused by general embryonic delay, because the number of somites in *RNF213* morphants and scrambled control morphants were equal.

Severely abnormal sprouting vessels were further seen in the head region. Large trunk arteries, such as the lateral dorsal aorta, basilar artery, and primordial channel formed almost normally. However, these vessels were of an irregular diameter and showed aberrant sprouting (Figure 9B). Remarkably, *RNF213* morphants sprouted abnormal vessels from the optic vessels at 60-72 hpf. In control morphants, the inner optic circle (IOC) was formed by a branch of the nasal ciliary artery; even at later stages, up to 7 days post-fertilization (dpf), three branches connected the lateral side of the eyeball to the IOC. However, in two different *RNF213* morphants, multiple vessels sprouted from the IOC and connected to the cranial veins (Figure 9C, red arrow, Figure S11).

## Discussion

To identify a causative gene for moyamoya disease, we used exome analysis in eight index cases who met the RCMJ criteria and whose families comprised three generations of patients. Thus, our cases and families can safely be assumed to be prototypical for familial moyamoya disease. For filtering we employed a modified version of Ng *et al.*'s criteria [29]. This modification was made to take account of the possible involvement of uncharacterized genes or the susceptibility gene. After applying this filtering process, we obtained two candidate genes, *PCMTD1* and *RNF213*. The former was discarded because it was not replicated by Sanger method in any of the eight index cases. However, p.R4810K in the latter showed complete disease segregation in all 42 families, confirming the previously identified genetic locus 17q25.3 [19]–[21]. Furthermore, we showed that p.R4810K was strongly associated with moyamoya disease (OR = 111.8) in all East Asian cases. Finally, we searched for variants in *RNF213* in non-p.R4810K East Asian cases and in Caucasian cases. We found five novel variants in seven Chinese cases and four variants in four Caucasian cases.

Additionally, we identified a founder haplotype and its decayed haplotypes in 42 families. Homogeneity of the haplotypes was confirmed by genotyping of 39 SNPs. These observations strongly suggest that the causative variant should be located within the core LD block region spanning from *RNF213* to *NPTX1*. However, complete sequencing of this region including promoter, intron and intergenic regions in one of the index cases did not show any variants other than p.R4810K or a G>A substitution in intron 11 of *FLJ35220*. The latter variant did not have any effect on splicing or gene expression levels. Furthermore we failed to detect any small Indel in exons in the eight index cases and large CNVs in three index cases in 17q25.3, although we missed small intronic structural abnormalities, which might be a possible cause of moyamoya disease. Taken together, these lines of evidence consistently support a conclusion that *RNF213* is a susceptibility gene for moyamoya disease. Recently a significant association of a SNP in *RNF213* with moyamoya disease in Japanese has been shown [33].

As we previously reported, the penetrance of autosomal dominant moyamoya disease is low, as illustrated by discordant identical twins or “skipping a generation” [34]. This characteristic of moyamoya disease is in accordance with the notion that *RNF213* is a susceptibility gene for moyamoya disease and reconciles our unexpected observation that this variant was found at an allele frequency of 1% in the Japanese, Korean and Chinese control populations. In addition, the low identification rates of *RNF213* variants in Caucasians compared with East Asians strongly suggests that there is genetic heterogeneity of moyamoya disease between these two populations. In addition, there are obvious differences in the variants themselves between East Asians and Caucasians. Further studies are needed to investigate whether such allelic differences result in the different clinical features.

By cloning the cDNA for this region, we discovered a novel splicing variant for *RNF213* (AB537889), which lacks exon 4 of *RNF213* [NM_020914.4]. This new splicing variant of *RNF213* is a major transcript of *RNF213*. We have experimentally proven that two functional domains, a Walker motif and a RING finger domain, function. Such novel features of *RNF213* hampered our elucidation of its physiological function.

To characterize the physiological role of *RNF213 in vivo*, we investigated the effects of *RNF213* suppression on zebrafish vasculature. *RNF213* knockdown zebrafish showed severely abnormal sprouting vessels in the head region, especially from the optic vessels at 60–72 hpf. In normal embryos, the overall wiring pattern of the major vessels is largely completed by 2–2.5 dpf, despite massive development of smaller-caliber vessels in the head through to 7 dpf [35]. Therefore, the aberrant vessel formation from the IOC seen in *RNF213* morphants at this stage implies a severely impaired program of angiogenesis in the head region. Although abnormal vascular phenotypes in the tail region have been reported after manipulation of genes such as plexin D1 [36], morphants showing aberrant vascular sprouting from the IOC have never been seen [37], suggesting that *RNF213* is involved in a novel signaling pathway in intracranial angiogenesis. As such, we expect that once we identify the pathway, we may be able to elucidate the consequences of vascular remodeling in moyamoya disease [9], [12], [23], [38]. We are aware of a limitation in the current study as a morpholino rescue experiment was not conducted. The rescue experiment is technically difficult to achieve because of the extremely large size of the *RNF213* mRNA. However, the similar and clearly defined vascular phenotypes shown by two different *RNF213* morphants indicate that these phenotypes were derived from the specific deletion of *RNF213* and not from an off-target effect of a morpholino.

A question remains as to how the p.R4810K variant or other nine variants may impair the physiological function of *RNF213* thereby resulting in moyamoya disease. We could not demonstrate that p.R4810K or p.D4013N affects ubiquitin ligase activity or causes other hallmark changes such as mRNA or protein instability. Furthermore, it appears that there was no gene dosage effect because some Japanese controls were homozygous (Table 2). In addition, homology search argued against pathological roles for some variants. It is thus probable that these variants likely perturb RNF213 function by unidentified mechanisms other than through ubiquitin ligase activity or by decreasing protein stability or by mislocalization. At present, we cannot specify the mechanism as haploinsufficiency, dominant negative or gain of function; instead we postulate the involvement of another factor. Requirement of co-factors with *RNF213* might also explain the lower disease prevalence (1 in 10,000) than expected from the relatively high minor allele frequency of approximately 1%. Furthermore, despite similar frequencies of p.R4810K, population attributable risks were different among the three East Asian populations, suggesting existence of environmental factors or another genetic factor. It should be noted, however, that involvement of the additional genetic factor is unlikely because the gene, *RNF213*, was singularly filtered as the common genetic factor by exome. We consider it rational to postulate involvement of environmental factors rather than genetic factors. Several environmental factors may include autoimmune conditions [39], [40], infection [41]–[45] and exposure to radiation [46]. Recently, Bauersachs *et al.* reported that *RNF213* is upregulated in bovine endometrium by pregnancy [47]. In addition, Zhang *et al.* reported that intracellular parasites and cytokines upregulate *RNF213* [48]. These findings support that *RNF213* is upregulated by inflammatory signals generated by interferon or cytokines. Of particular interest is transforming growth factor beta (TGF-β), because it is known to be elevated in the cerebrospinal fluid, blood and arteries of patients with moyamoya disease [23], [38] and a SNP within TGF-β has been reported to be associated with moyamoya disease [49]. Further study on cross-talk of *RNF213* with such conditions that cause endothelial damage, or angiogenesis factors is needed to understand the molecular mechanisms.

Sharing p.R4810K among moyamoya cases urged us to discuss the anthropological history of the founder haplotype carrying p.R4810K. p.R4810K appears to be a neutral variant or an advantageous variant for human survival because it has been maintained in the East Asian population. It is interesting that p.R4810K was not found in Caucasians; the relatively high prevalence of p.R4810K among East Asians could account for the higher prevalence of moyamoya disease in East Asians than in Caucasians.

This study has several limitations. First, we cannot provide evidence regarding impairment of physiological function of these variants of *RNF213* in moyamoya disease. Second, the number of subjects used for deep sequencing or CNVs was small. The strengths of our study are that we conducted whole genome-exome analysis and demonstrated strong evidence showing involvement of a single gene, *RFN213*, in moyamoya disease. Other strengths include that we found a founder variant in East Asian cases, an additional nine variants in Chinese and Caucasian cases, and characterized the RNF213 protein biochemically and physiologically. Although further studies are necessary to clarify the biochemical function and pathological role of *RNF213* in moyamoya disease, the discoveries of its association with the disease and its unique roles in angiogenesis may pave a way to early diagnosis and prevention. It should be noted, however, that the majority of the pathological proof awaits further studies.

## Supporting Information

Text S1 Supplemental methods, reference and acknowledgments.
(DOC)

Appendix S1 Web Resources.
(DOC)

Figure S1 Pedigree chart. Forty-one Japanese families and one Korean (pedigree 40) family participated in this study. The phenotypes of occlusive lesions are shown. Genotype of p.R4810K (G>A) is shown in brackets.
(TIF)

Figure S2 A variant in a Caucasian family. The index case is the father (CAU_ped1_12), who was born in 1966 and suffered a mild ischemic stroke at the age of 30. His mother died of an ischemic stroke at the age of 35. He has four children from two marriages. The second child from the first marriage (CAU_Ped1_122) was born in 1991 and developed moyamoya disease at the age of 5. The first child of the second marriage (CAU_Ped1_123) was born in 1999 and developed moyamoya disease with symptoms of involuntary movement at the age of 9. The second child, (CAU_Ped1_124) born in 2006, developed moyamoya disease with manifestations of ischemic stroke at the age of 3. Diagnoses of moyamoya disease were made by magnetic resonance imaging (MRI). (A) Caucasian pedigree. The index case (father) and his three affected children carry the p.D4013N variant of *RNF213* (G>A). Genotypes of the variant for each member are shown in the bracket in the pedigree. (B) Sequencing analysis. (C) Genotyping by HpyCH4V.
(TIF)

Figure S3 Effect of G>A substitution in intron 11 of *FLJ35220* on splicing or gene expression. (A) We tested whether exon 11 was read through. A short form, which skips exon 11, had an expected size of 107 bp. A long form, which reads through exon 11, had an expected size of 166 bp (NM_173627.2). M, 100 bp ladder DNA marker. (B) *FLJ35220* mRNA expression in LCLs [controls; JPN1, an unaffected daughter (Ped17_123) of Ped17_12 and an unaffected spouse (Ped18_20) of Ped18_2; cases: Ped11_11, Ped17_12, Ped18_2 and Ped18_22] as determined by real-time quantitative PCR. Data are shown as means ± S.D. of three independent experiments. There is no statistically significant difference between the two groups. Significance was tested using Student's *t*-test. A *p*<0.05 was considered to be significant. The methods have been fully described in the Text S1.
(TIF)

Figure S4 Copy number analysis for the 1.5-Mb region in 17q25.3. Three index cases of pedigrees 5, 11 and 18 were analyzed. The blue lines represent the copy numbers (log 2 ratio) averaged over 10 SNPs. The copy numbers were compared with control spouse of 2 of pedigree 18.
(TIF)

Figure S5 Sequence chromatograms for the eight novel variants.
(TIF)

Figure S6 Cloning of full-length *RNF213* cDNA. Schematic representation of the internal connecting site of *RNF213* cDNA. RT-PCR with primers set on *RNF213* resulted in amplification of the expected fragments. First, fragments 1, 2, 4, and 5 were cloned into a pcDNA3.1+ vector. Second, fragment 3 was cloned into the vector carrying fragment 2, and fragment 5 was subcloned into the vector carrying fragment 4. Then fragments 2–3 and 4–5 were subcloned into fragment 1 using the restriction enzyme sites indicated.
(TIF)

Figure S7 Northern blotting analysis of *RNF213* mRNA. (A) *RNF213* mRNA expression in the indicated human tissues (heart, brain, liver, pancreas, skeletal muscle, and lung). Arrow indicates *RNF213* mRNA. (B) *RNF213* mRNA expression in cultured human cells (HeLa, HEK293T and LCLs (control: JPN1, Ped18_20 (a spouse of 2) and case: Ped18_2, Ped18_22, Ped11_11)) using radiolabelled probes corresponding to *RNF213* coding regions. *GAPDH* mRNA expression is shown as a loading control. The position of RNA Millennium markers (Ambion) and positions of the 18S and 28S ribosomal RNAs are indicated on the left.
(TIF)

Figure S8 Characterization of the p.R4810K and p.D4013N allele proteins of *RNF213*. (A) Stability of the p.R4810K variant. Wild-type or p.R4810K variant of RNF213-HA were transiently expressed in HEK293 cells. Cells were lysed and subjected to immunoblotting with an anti-HA antibody. IP, immunoprecipitation. IB, immunoblot. (B) Subcellular localization of the p.R4810K variant of RNF213. HEK293 cells transiently expressing the wild-type or p.R4810K variant of RNF213-HA were fractionated into cytosol, membrane/organelle, nucleus, and cytoskeleton using different lysis reagents (ProteoExtract kit, Calbiochem). (C) Self-ubiquitination of the p.R4810K variant of *RNF213*. HEK293 cells transiently expressing the wild-type or R4810K variant of RNF213-HA and Myc-ubiquitin were lysed and subjected to immunoprecipitation using an anti-HA antibody, followed by immunoblotting using an anti-Myc antibody. (D) Self-ubiquitination of the p.D4013N mutant of *RNF213*. HEK293 cells transiently expressing the wild-type or p.D4013N mutant of RNF213-Flag and Myc-ubiquitin were lysed and subjected to immunoprecipitation using an anti-Flag antibody, followed by immunoblotting using an anti-Myc antibody.
(TIF)

Figure S9 Allele-specific mRNA expression of *RNF213* by labeling and detection of the two alleles of marker SNPs p.R4810K and p.H4557H. (A) Design of allele-specific mRNA expression using SNaPshot assay. Arrows indicate primer positions for amplification. Bold arrows indicate extension primers for each SNP. (B) Genotypes of *RNF213* gene at two SNPs (p.H4557H and p.R4810K) in cases for SNaPshot assay. (C) Allele-specific ratio of *RNF213* mRNA expression in LCLs from SNP heterozygous patients. The common allele/rare allele ratio from cDNA was normalized to that ratio from genomic DNA of the same individual. Data are shown as means ± S.D. of three independent experiments. There was no statistically significant difference between the two alleles for each SNP. Significance was tested by Student's *t*-test. A *p*<0.05 was considered to be significant.
(TIF)

Figure S10 Splicing ablation of *RNF213* transcripts by morpholino injection. RT-PCR showing the defective splicing induced by *RNF213*-*α*-MO1 and MO2 pairs and a *RNF213*-*β*-MO. Compared with the results of PCR (using primer pairs *RNF213*-*α*_2 and 2R, *RNF213*-*β*_3 and 3R, and *RNF213*-*β*_1 and 1R) from noninjected embryos, where a single band was generated (lanes 1, 3, and 5, marked “native”), split bands were detected in PCR using embryos injected with a *RNF213*-*α*-MO1, MO2 pair and a *RNF213*-*β*-MO (lanes 2, 4, and 6, marked “exon blocked”). M, 1-kb ladder DNA marker. Band indicated by ‘genomic’ is the amplified genomic sequence. Equal amounts of PCR products and marker were loaded in each lane. Intensity of RT-PCR products indicates that *RNF213*-*α* is dominantly expressed in vivo.
(TIF)

Figure S11 *RNF213* morphants show multiple sprouting vessels from IOC. Bar graphs showing the number of sprouting vessels from IOC of Tg(fli-EGFP)y1 embryos 72 h post-fertilization. Each group was injected with 2.5 ng morpholinos (MO1, MO2) per embryo indicated in each lane. *n* = 20 per group. Values are means ± SD. *p<0.01 versus control scramble morphants. #*p*<0.01 versus *RNF213*-*β*-morphants. Neither group of controls nor *RNF213*-*β* showed any extra sprouting vessels.
(TIF)

Table S1 Demographic feature of familial participants.
(XLS)

Table S2 Summary of demographic and clinical profiles of cases and controls.
(XLS)

Table S3 Primers used for amplification of *RNF213* and *PCMTD1* (Human build 37.1).
(XLS)

Table S4 Positional candidate genes in the 1.5 Mb locus on 17q25.3 (Map Viewer: Build 37.1).
(XLS)

Table S5 Primer sets and restriction enzymes for screening variants in controls.
(XLS)

Table S6 Primers used for SNaPshot assay.
(XLS)

Table S7 Numbers of variants and candidate genes in the 2nd stage for various combination subsets of cases.
(XLS)

Table S8 A summary of the sequencing results for five controls by exome, 10 controls and deep sequencing in a control by the Sanger method.
(XLS)

Table S9 Association of ss179362673 with moyamoya disease with or without family histories by allelic model.
(XLS)
