# Supplementary material for: Identification of RNF213 as a Susceptibility Gene for Moyamoya Disease and Its Possible Role in Vascular Development
Source: PLoS One. 2011 Jul 20;6(7):e22542. doi: 10.1371/journal.pone.0022542 (PMC3140517; doi:10.1371/journal.pone.0022542)
Supplement: Text S1 — Supplemental methods, reference and acknowledgments. (DOC) [file pone.0022542.s001.doc]

**Text S1** Supplemental methods, reference and acknowledgments

**Supplemental methods**

Bacterial Artificial Chromosome (BAC) clones and shotgun sequencing

Two BAC contigs were generated to cover long intergenic regions: one between nucleotides 78194200 and 78238718, covering solute carrier family 26, member 11 (*SLC26A11*) and the 5′ end of *RNF213*, and the other between nucleotides 78345170 and 78450404, covering *RNF213*, hypothetical protein FLJ35220 (*FLJ35220*) and neuronal pentraxin 1 (*NPTX1*) (UCSC Genome Browser in Appendix S1). The sequences of two BAC clones were determined by a shotgun approach [1,2].

Southern blotting

Genomic DNA (10 μg) isolated from lymphoblastoid cell lines (LCLs; described below) was Southern blotted. A probe covering exons 12 to 20 (1084 bp) was PCR amplified from a plasmid containing the *RNF213* cDNA with the primers 5′-TCACCG TTCCGGGAACAA-3′ and 5′-ACACAGAGTCGGCAACAGCTAT-3′ and was labeled with ^32^P-dCTP.

39 SNPs

We selected five rare variants and 34 SNPs for typing to cover the region around *RNF213*. The 39 SNPs selected were rs6565649, rs7216577, rs7406843, rs8078855, rs7217421, rs9902702, rs11869363, rs12451808, ss179362670, ss179362671, rs55996424, rs7222014, rs35968416, rs4890012, rs12150356, ss179362672, rs8070106, rs4889848, ss179362673, rs6565683, rs9913006, rs6565686, ss179362674, rs8065843, rs4074303, rs4890025, ss179362675, rs11869626, rs9898443, rs12601738, rs12185227, rs7502866, ss161110142, rs9911978, rs12950635, rs4890047, rs4889863, rs11655474 and rs8080957 (dbSNP and Hapmap database in Appendix S1).

Screening variants by restriction enzyme fragment length polymorphisms (RFLP) in controls

The PCRs were performed with 30 ng genomic DNA, LA Taq DNA polymerase (Takara), 2.5 mM of each dNTP and 0.3 μM of the forward and reverse primers (Table S5). The cycle conditions were 95°C for 9 min followed by 40 cycles of 94°C for 45 s, a given annealing temperature for each primer (Table S5) for 45 s, 74°C for 1 min then a final extension at 72°C for 7 min. After PCR amplification, variants were identified by RFLP as described in Table S5 with various restriction enzymes (New England Biolabs, Inc., Tokyo) for 3 h and electrophoresed on agarose gels.

Cloning of *RNF213* and construction of expression vectors

Five fragments of *RNF213* (Figure S6) were amplified by RT-PCR from HEK293 RNA using the SuperScript III One-Step RT-PCR System with Platinum Taq DNA Polymerase (Invitrogen) with the primers described below. Fragment 1: 1CloF RNF213 (5’-ATG CGC TAG CCG CCA TGG AGT GTC CTT CGT GCC AGC ATG TCT CC-3’) and 2CloR RNF213 (5’-CGG AAT TCG TGC ACA TGA TCT TAA GTT CTG AAT CCA GGT CCG-3’). Fragment 2: 3CloF RNF213 (5’-GCT CTA GAC AGA ACT TAA GAT CAT GTG CAC CGT GGA CCA CC-3’) and 3CloR RNF213 (5’-CGG GAT CCC TCG AGG GGT GCC TGG GGG GTG ACG AAC GCC-3’). Fragment 3: 4CloF RNF213 (5’-GCT CTA GAC TCG AGG CCA TCC AAG CCT ACC TGG CAG GTC ACT ACC-3’) and 4CloR RNF213 (5’-GAA AGA TGT CCA AAG CTT GGA TGT CAT CCT TGC C-3’). Fragment 4: 5CloF RNF213 (5’-AGG ATG ACA TCC AAG CTT TGG ACA TCT TTC CTG GCC-3’) and 5CloR RNF213 (5’-GAG AGA ATT CGT CTG GCA AGG CCA GTT AAA CAG TAG GGG C-3’). Fragment 5: 6CloF RNF213 (5’-CAG ACG AAT TCT CTC CAG CTG TTT CCC AAG CGC-3’) and 6CloR RNF213 (5’-AAG GAA AAA AGC GGC CGC TAT CTC ATT TCT CGA TTC CAT TTC AGC ACA GCA GC-3’).

The contiguous arrangement of the fragments to form the full-length *RNF213* cDNA is shown in Figure S6. Fragments 1 and 2 were connected using the *Apa*LI site at bp 3972–3977. Fragments 2 and 3 were connected using the *Xho*I site at bp 5866–5871. Fragments 3 and 4 were connected using the *Hind*III site at bp 8975–8980. Fragments 4 and 5 were connected using the *Eco*RI site at bp 12118–12123. The full-length cDNA of *RNF213* was subcloned into the pcDNA3.1+ expression vector with exogenously added *Nhe*I and *Not*I sites. It should be noted that the section consisting of fragments 2 and 3 contains three *Apa*LI sites, so this section was partially digested by *Apa*LI for 30 min, then the fragment in which only the 5’ terminal *Apa*LI site was digested was selected and used for ligation with fragment 1. Hemagglutinin (HA) or Flag tags were fused to the C-terminus of the *RNF213* cDNA by PCR using the primers 6CloR*RNF213*HA (5’-AAG GAA AAA AGC GGC CGC TAA GCG TAG TCT GGG ACG TCG TAT GGG TAT CTC ATT TCT CGA TTC CAT TTC AGC ACA GCA GC-3’) or 6CloR*RNF213*Flag (5’-AAG GAA AAA AGC GGC CGC TAC TTA TCG TCG TCA TCC TTG TAA TCT CTC ATT TCT CGA TTC CAT TTC AGC ACA GCA GC-3’), respectively.

To generate a RING finger-deleted mutant of *RNF213*, an exogenous *Eco*RI site was fused to the 3’ terminus just before the RING finger domain. This site was then ligated to an endogenous *Eco*RI site located just after the RING finger, enabling the RING finger domain to be skipped from the *RNF213* cDNA.

Detection of splicing products of *RNF213* cDNA or *FLJ35220* cDNA and real-time quantitative PCR

The PCR primers used were *RNF213* ex3F (5’-CAA TAA CTC CAC AAT GGC GT-3’) and *RNF213* ex4R (5’-GGG TTT GAA AGC AAA GTC AGG-3’). The expected product size was 417 bp for NM_020914.4 and 270 bp for AB537889, respectively. The PCR primers used were *FLJ35220* cex10F (5’-TGA CAT CTG CTC CCG AGA G-3’) and *FLJ35220* cex12R (5’-ATG AGA CGA GGA CGT GTG CT-3’). The expected product sizes were 166 bp for NM_173627.2.

Quantitative PCR for *RNF213* cDNA was performed using primers *RNF213* cex51F (5’-CAC GCC AGA GCA ATG TGA A-3’) and *RNF213* cex52R (5’-TCA AGG TTG CTG TCA CTA GGC C-3’). Quantitative PCR for *FLJ35220* cDNA was performed using primers *FLJ35220* cex7F (5’-ACC TTG GCG TCC TTA CAG ACC T-3’) and *FLJ35220* cex8R (5’-CAG GAG TCG GAT CTT CTC CTT G-3’). Target cDNA was normalized to the expression levels of a reference gene, peptidyl-prolyl isomerase A (*PPIA*) [3].

Northern blotting

The two probes *RNF213*_1 (492 bp) and *RNF213*_2 (591 bp) were amplified by PCR from a full-length *RNF213* plasmid with primers: 5’-GTC ACC TGG TTA TGT ATA TGG AAA AC-3’ and 5’-TGT GAA TGA GGC ACC TGA AGA T-3’; and 5’-ATC TCA TCA GCC AAG ATA AGC GTA T-3’ and 5’-CGG CGT AGA CAA TTT CAT TGT GTA-3’, respectively. The glyceraldehyde-3-phosphate dehydrogenase (*GAPDH*) probe corresponded to positions 626–1078 of human *GAPDH* cDNA.

Rapid amplification of cDNA ends (RACE)

The 5’ cap structure was removed and the GeneRacer RNA Oligo (5’-CGA CUG GAG CAC GAG GAC ACU GAC AUG GAC UGA AGG AGU AGA AA-3’) was ligated to the 5’ end of the mRNA. The cDNA was obtained from the full-length mRNA by RT-PCR using a GeneRacer Oligo dT primer (5’-GCT GTC AAC GAT ACG CTA CGT AAC GGC ATG ACA GTG (T)^24^-3’). Using this cDNA as a template, the 5’ end of the *RNF213* cDNA was amplified using the GeneRacer 5 primer (5’-CGA CTG GAG CAC GAG GAC ACT GA-3’) and a gene-specific reverse primer (5’-GCC TCC CAC TTT ACC TCA CAC CCG-3’). Likewise, the 3’ end of the *RNF213* cDNA was obtained using a gene-specific forward primer (5’-GTG TAG GGT GAG CGT AAT GTT TTG T-3’) and the GeneRacer 3 primer (5’-GCT GTC AAC GAT ACG CTA CGT AAC G-3’). The PCR products were cloned into the pCRII Blunt-TOPO vector (Invitrogen) and then sequenced using vector-specific primers.

Immunostaining and subcellular fractionation

Cells transiently expressing HA-tagged RNF213 were fixed with 4% paraformaldehyde for 15 min, washed twice in phosphate-buffered saline (PBS) containing 0.2% Triton X100, permeabilized and blocked in PBS containing 0.2% Triton X100, 1% bovine serum albumin, 1% goat serum and 20% glycerol for 1 h. Then an anti-HA antibody was added to the blocking solution, and the cells were incubated for a further 1 h. The cells were washed twice in PBS containing 0.2% Triton X100, and incubated with Alexa Fluor 488-conjugated anti-mouse IgG (Invitrogen, Tokyo) for 1 h. Fluorescent signals were analyzed with an LSM 510 META confocal laser microscope (Carl Zeiss). The subcellular distribution of *RNF213* was determined using a ProteoExtract Subcellular Proteome Extraction kit (CALBIOCHEM), which contains four different lysis buffers for sequential lysis of cells, in accordance with the manufacturer’s instructions.

ATPase Assay

The ATPase assay was performed using a BIOMOL Green kit (BIOMOL) in accordance with the manufacturer’s instructions. Briefly, the GST-tagged fragment, at a final concentration of 0.02 mg/mL, was incubated in buffer containing 5 mM ATP, 50 mM HEPES-KOH pH 7.5, 300 mM KCl and 10 mM MgCl_2_ at 37°C for the indicated times. The reaction was stopped by adding perchloric acid to a final concentration of 1%, and was incubated with Malachite Green solution (BIOMOL Green; BIOMOL) at room temperature for 20 min, after which the absorbance at 630 nm was measured. The amount of released phosphate was estimated by comparison with the absorbance of a mixture of control free phosphate and BIOMOL Green.

Zebrafish model

The overwhelming majority of MOs elicited a robust and specific effect, which was dose-dependent in either the strength of the phenotype or the penetrance, for moderate (< 5 ng/embryo) and high (6–9 ng/embryo) doses [4]. Higher doses of z*RNF213* MO resulted in a larger average reduction of z*RNF213* gene expression, but also caused detectable detrimental effects on development. Therefore, an effective dose of 2.5 ng was determined for these MOs. The following MOs were used: *RNF213-α−*MO1-A (5’-ACT CGT TGA TGT CTG AAG TGA TAA A-3’), *RNF213-α*-MO1-D (5’-AGC TAG GAG AAA GTC CTA CCA ATT T-3’), *RNF213-*α*−*MO2-A, (5’-ATC TTC CTA ATA AAG AGT TAG AAC A-3’), *RNF213-*α*−*MO2-D, (5’-AGT AAT GGA AAG ACT CTT ACC TGG C-3’) and *RNF213-*β*−*MO-D (5’-GGT ATA ACT AGT ACT TAC CAA CAG C-3’). The 5-bp-mismatched MOs *RNF213-*α*−*MO1-Dc (5’-AGG TAC GAG AAA CTC CTA GCA ATT T-3’) and *RNF213-*α*−*MO1-Ac (5’-AGT GCT TCA TGT CTG AAC TGA TAA A-3’) were used as controls. To determine efficacy levels, reverse transcription was performed with 2.0 μg of total RNA isolated from wild-type embryos and embryos injected with each MO at 48 h post-fertilization (hpf) using random oligo primers (1 μg). The following cycle parameters were used for PCR: 94°C for 2 min; 30 cycles of 94°C for 30 s, 60°C for 40 s and 72°C for 2 min; then 72°C for 5 min. The following primers were used for MO efficacy experiments: *RNF213-*α_2 (5’-GTC AGC AAA GGT GGT CAT GAT GGT CAA AGA GAC-3’), *RNF213-*α_2R (5’-CAG AAG CGT AGG CAG CTC TCC ATT TTC TTA CAG A-3’), *RNF213-*α_3 (5’-ATG CTC TAT GTG AAC CAA CTC AAC TAT GAA GT-3’), *RNF213-*α_3R (5’-CGG AAC TCT TGG TCA TCC ATA AGG GGA TCA T-3’), *RNF213-*β_1 (5’-GGT TTG GAC AAC ATC TTT TAC GTG AAG GTG A-3’ and *RNF213-*β_1R (5’-CCT TTG AAC TTC TCA AAA AGG CGT CTC ACA T-3’). For imaging, confocal microscopy of animals was performed on a Leica TCS-SP5 confocal microscope with a krypton–argon laser source. Animals were treated with 6-propyl-2-thiouracil to inhibit pigment formation and were mounted for imaging in 2% agarose in E3 solution with tricaine/MS-222, as per previously published methods [5]. Image stacks were collected with 7-μm spacing between the planes. Image rendering from 40 collected image stacks was performed with LAS AF software (Leica) in Appendix S1.

**Supplemental References**

1. Gordon D, Abajian C, Green P (1998) Consed: a graphical tool for sequence finishing. Genome Res 8: 195-202.

2. McMurray AA, Sulston JE, Quail MA (1998) Short-insert libraries as a method of problem solving in genome sequencing. Genome Res 8: 562-566.

3. Seitz U, Wagner M, Neumaier B, Wawra E, Glatting G, et al. (2002) Evaluation of pyrimidine metabolising enzymes and in vitro uptake of 3'-[(18)F]fluoro-3'-deoxythymidine ([(18)F]FLT) in pancreatic cancer cell lines. Eur J Nucl Med Mol Imaging 29: 1174-1181.

4. Nasevicius A, Ekker SC (2000) Effective targeted gene 'knockdown' in zebrafish. Nat Genet 26: 216-220.

5. Weintraub WS (2007) The pathophysiology and burden of restenosis. Am J Cardiol 100: 3K-9K.

**Supplemental Acknowledgments**

We thank the members of the Moyamoya Consortium for patient recruitment and help in performing MRI and MRA examinations: Hidetosi Ikeda (Department of Neurosurgery, Minami Tohoku Hospital); Ariiku Taki and Yashuhiko Wada (Kansai Rousai Hospital); Akira Handa and Hiroyasu Akutsu (Okayama Rosai Hospital); Hajime Touho (Touho Neurosurgery Clinic); Hidenori Miyake and Yasushi Ueno (Hamamatsu Rosai Hospital); Hidetoshi Nogaki (Wadayama Hospital); Junji Kitamura (Kitamura Clinic); Kiyohiro Houkin and Toshiyuki Onda (Sapporo Medical University Hospital); Takenaka Katunobu (Takayama Red Cross Hospital); Kunio Hashimoto (Tsuchiura Kyodo General Hospital); Makoto Sonobe (Mito Medical Center); Mitsuru Kimura (Nishiwaki Municipal Hospital); Noboru Kusaka (Iwakuni National Hospital); Nobuyuki Sakai (Kobe City General Hospital); Sadahiko Ban (Kobe City Public Health Center); Satoshi Kuroda (Hokkaido University Graduate School of Medicine); Shigetoshi Takaya (Tango Central Hospital); Tatsuhito Yamagami (Kizugawa Hospital); Yoshihiro Ohyama (Akishima Sougo Hospital); Yoshihiko Kamimura (Sakaide Kaisei Hospital); Yukio Wakuta (Saiseikai Yamaguchi General Hospital); Shinsuke Tominaga, Hiroshi Hasegawa, Toshihiko Inui, and Munenori Nagashima (Tominaga Hospital); Keisuke Yamada and Yo Kishi (Kyoto University Hospital); and Shigeki Yamada (Shiga Medical Center for Adults): Kyoto University has applied to the Patent Office, Japan, for a patent on this study.
