# Supplementary material for: Identification of RNF213 as a Susceptibility Gene for Moyamoya Disease and Its Possible Role in Vascular Development
Source: PLoS One. 2011 Jul 20;6(7):e22542. doi: 10.1371/journal.pone.0022542 (PMC3140517; doi:10.1371/journal.pone.0022542)
Supplement: Appendix S1 — Web Resources. (DOC) [file pone.0022542.s002.doc]

**Appendix S1** Web Resources

The URLs for data presented herein are as following:

Online Mendelian Inheritance in Man (OMIM), http://www.ncbi.nlm.nih.gov/Omim/

PROLIGO, http://www.sigmaaldrich.com/japan.html

NCBI, http://www.ncbi.nlm.nih.gov/

dbSNP, build 131, http://www.ncbi.nlm.nih.gov/projects/SNP/snp_summary.cgi?build_id%BC131

Hapmap database, http://hapmap.ncbi.nlm.nih.gov/

Golden Helix Software, http://www.goldenhelix.com

GENEHUNTER (Ver2.1_r6), http://www.staff.uni-marburg.de/~strauchk/software.html

BLAST, http://blast.ncbi.nlm.nih.gov/Blast.cgi

Leica LAS AF software, http://microscopy.duke.edu/analysis.html

RefSeq, http://www.ncbi.nlm.nih.gov/RefSeq/

SAMtools, http://samtools.sourceforge.net/

Genecard, http://www.genecards.org/

UCSC Genome Browser: http://www.genome.ucsc.edu
