# Supplementary figures and images for: Identification of RNF213 as a Susceptibility Gene for Moyamoya Disease and Its Possible Role in Vascular Development
Source: PLoS One. 2011 Jul 20;6(7):e22542. doi: 10.1371/journal.pone.0022542 (PMC3140517; doi:10.1371/journal.pone.0022542)

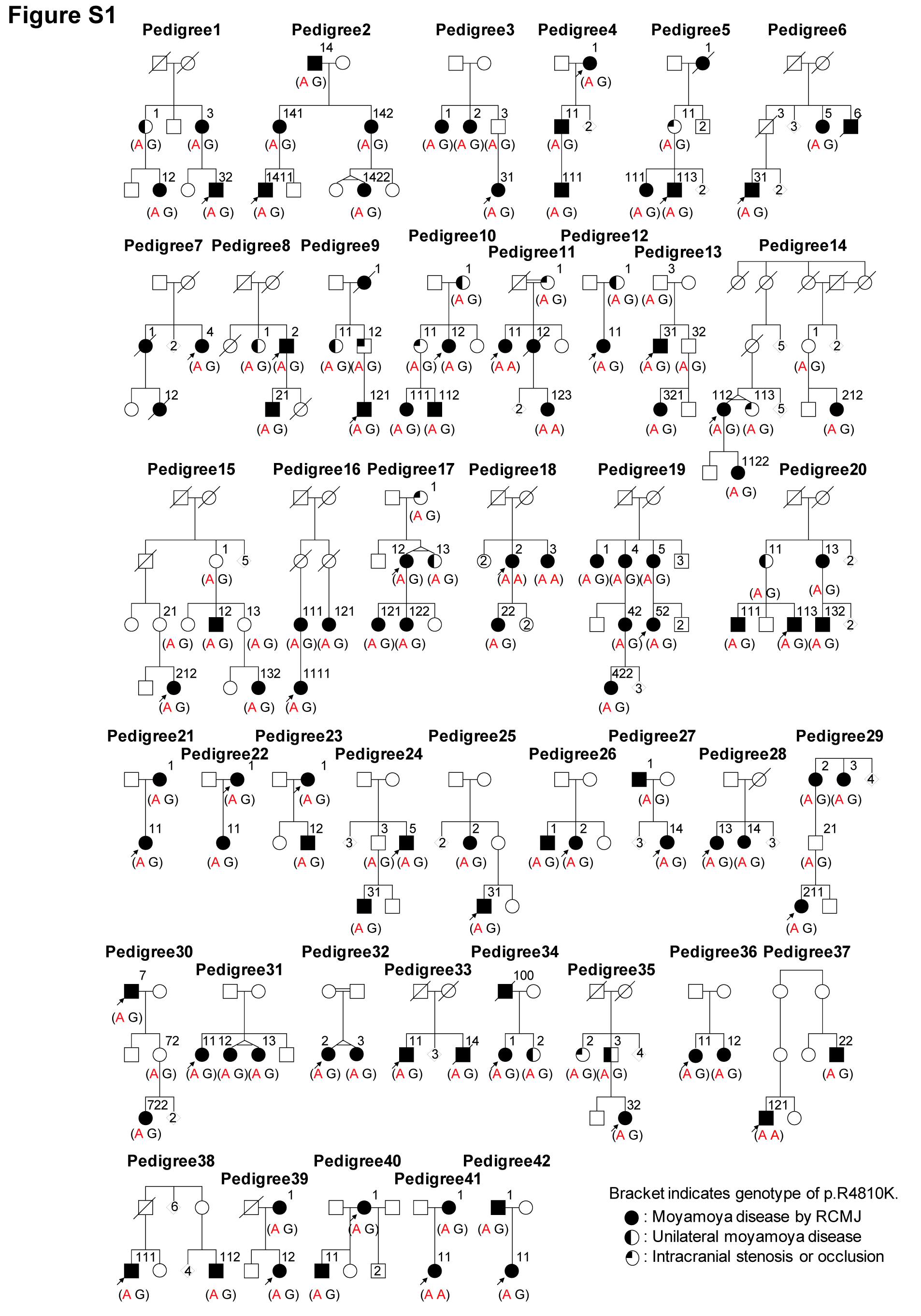

Supplement: Figure S1 — Pedigree chart. Forty-one Japanese families and one Korean (pedigree 40) family participated in this study. The phenotypes of occlusive lesions are shown. Genotype of p.R4810K (G>A) is shown in brackets. (TIF) [file pone.0022542.s003.tif]

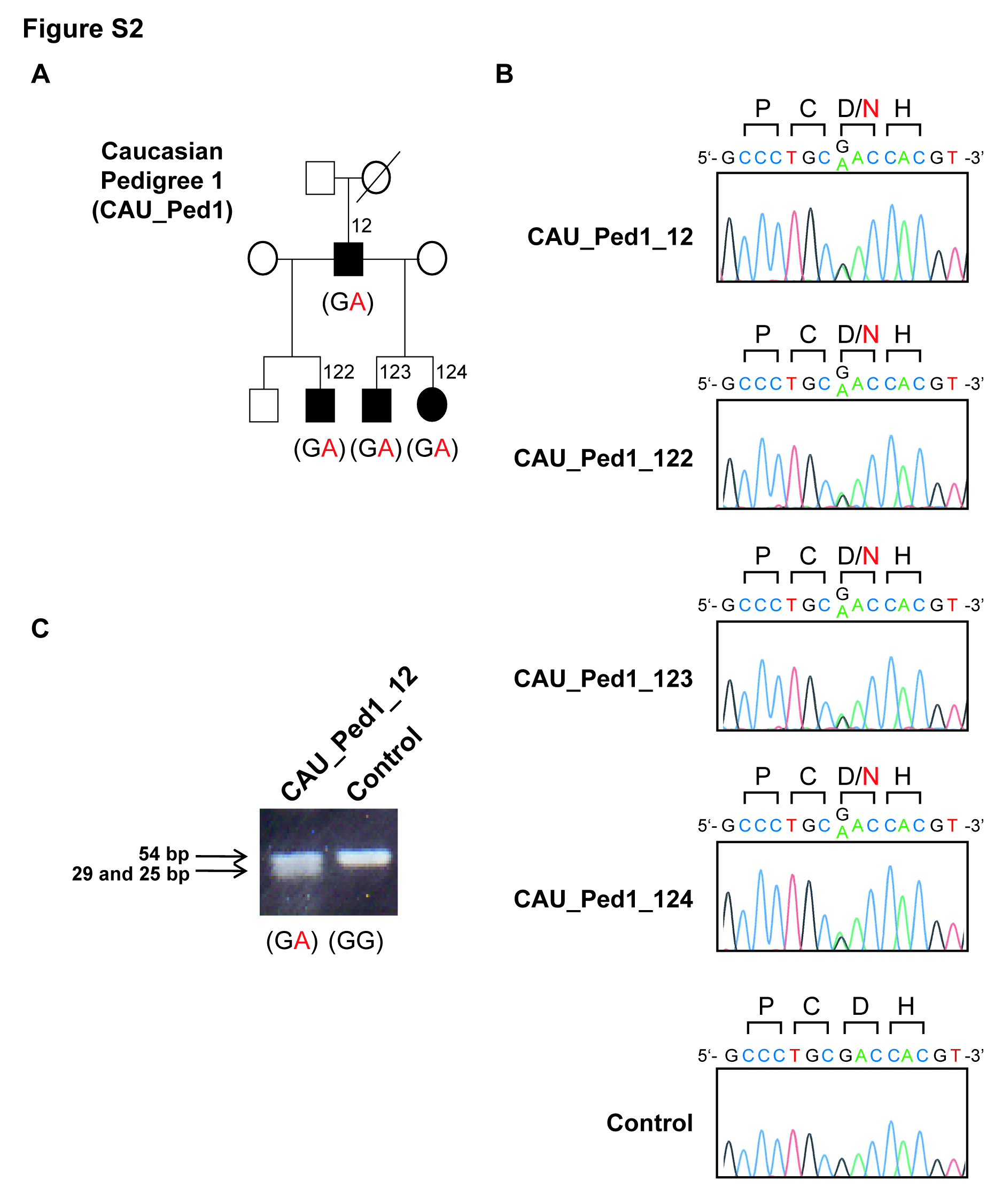

Supplement: Figure S2 — A variant in a Caucasian family. The index case is the father (CAU_ped1_12), who was born in 1966 and suffered a mild ischemic stroke at the age of 30. His mother died of an ischemic stroke at the age of 35. He has four children from two marriages. The second child from the first marriage (CAU_Ped1_122) was born in 1991 and developed moyamoya disease at the age of 5. The first child of the second marriage (CAU_Ped1_123) was born in 1999 and developed moyamoya disease with symptoms of involuntary movement at the age of 9. The second child, (CAU_Ped1_124) born in 2006, developed moyamoya disease with manifestations of ischemic stroke at the age of 3. Diagnoses of moyamoya disease were made by magnetic resonance imaging (MRI). (A) Caucasian pedigree. The index case (father) and his three affected children carry the p.D4013N variant of RNF213 (G>A). Genotypes of the variant for each member are shown in the bracket in the pedigree. (B) Sequencing analysis. (C) Genotyping by HpyCH4V. (TIF) [file pone.0022542.s004.tif]

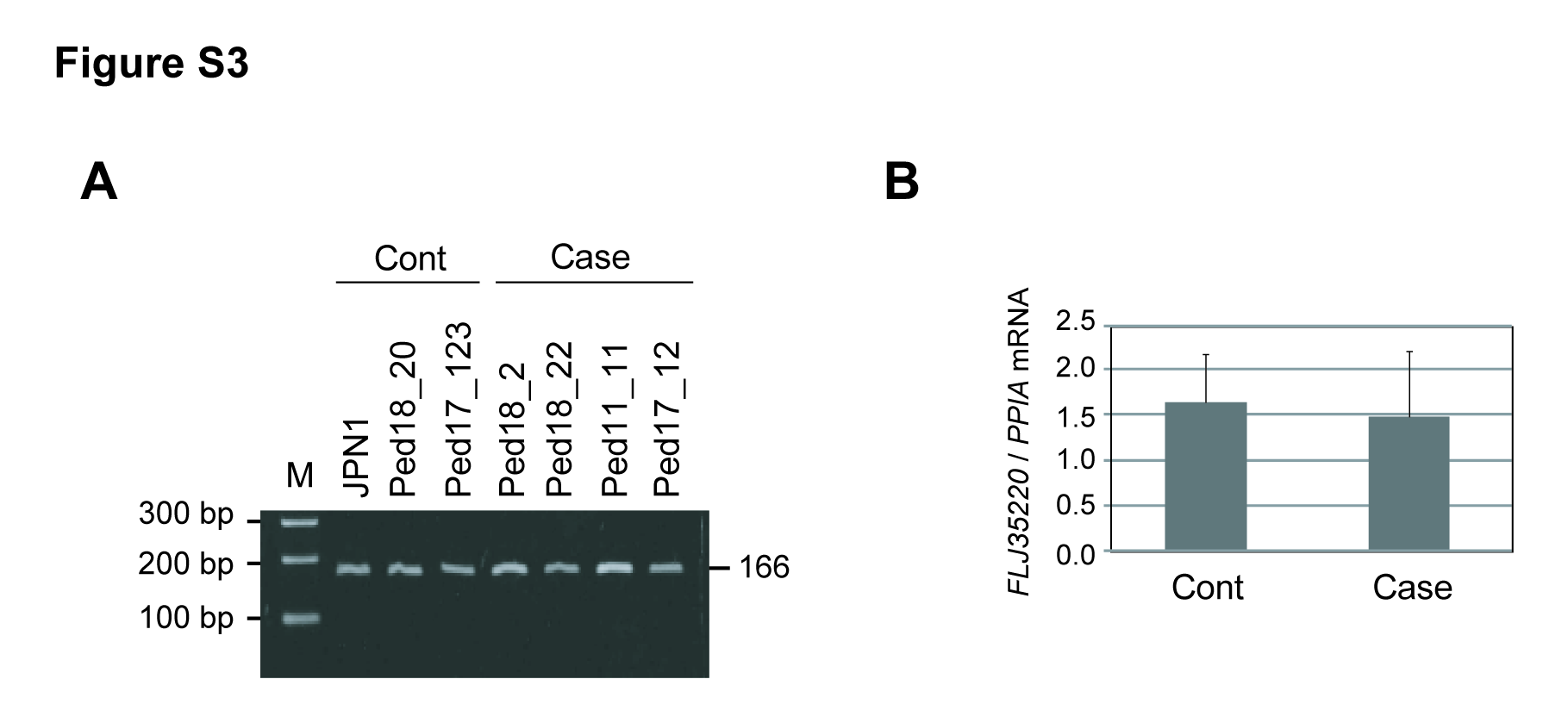

Supplement: Figure S3 — Effect of G>A substitution in intron 11 of FLJ35220 on splicing or gene expression. (A) We tested whether exon 11 was read through. A short form, which skips exon 11, had an expected size of 107 bp. A long form, which reads through exon 11, had an expected size of 166 bp (NM_173627.2). M, 100 bp ladder DNA marker. (B) FLJ35220 mRNA expression in LCLs [controls; JPN1, an unaffected daughter (Ped17_123) of Ped17_12 and an unaffected spouse (Ped18_20) of Ped18_2; cases: Ped11_11, Ped17_12, Ped18_2 and Ped18_22] as determined by real-time quantitative PCR. Data are shown as means ± S.D. of three independent experiments. There is no statistically significant difference between the two groups. Significance was tested using Student's t-test. A p<0.05 was considered to be significant. The methods have been fully described in the Text S1. (TIF) [file pone.0022542.s005.tif]

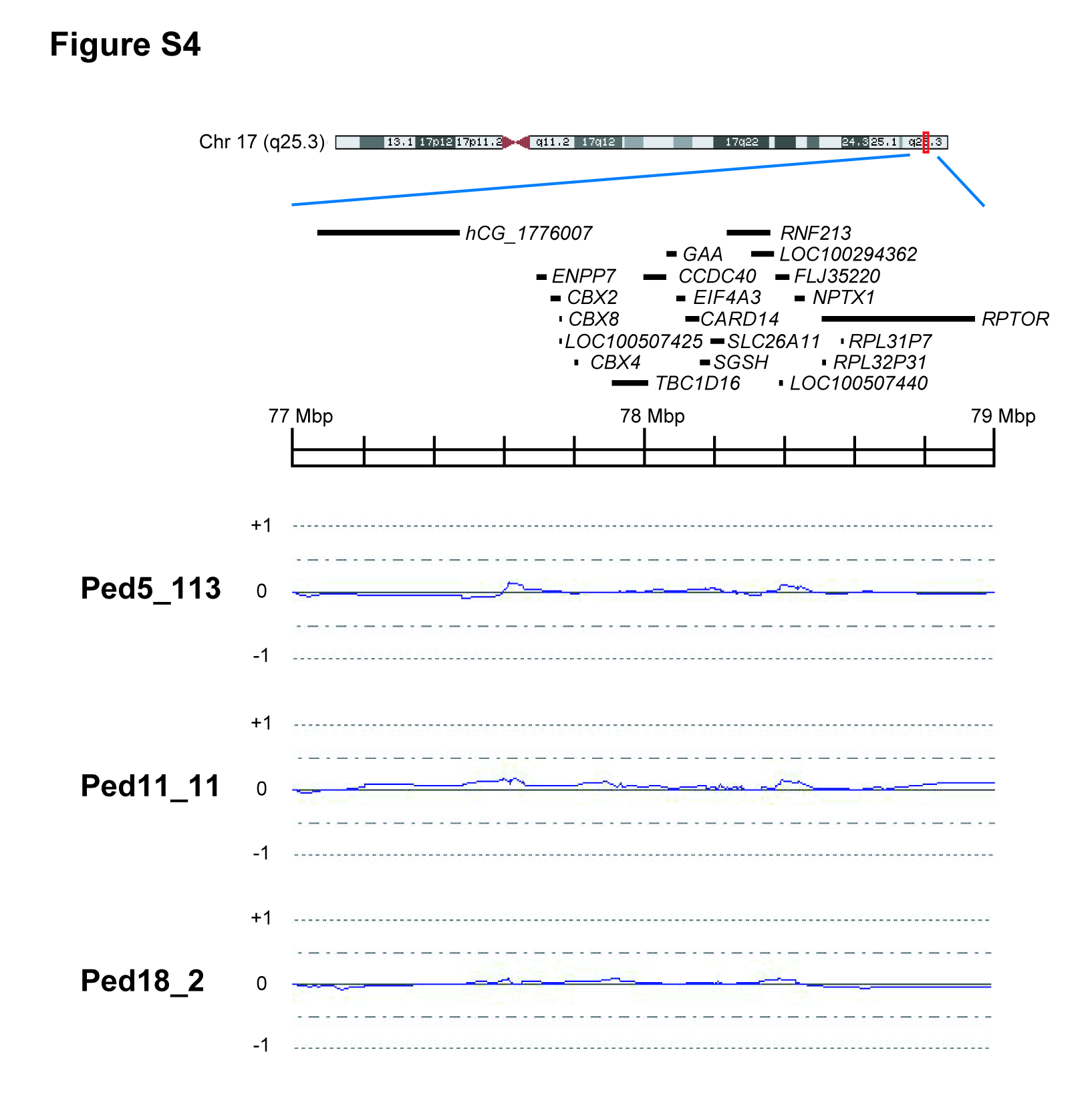

Supplement: Figure S4 — Copy number analysis for the 1.5-Mb region in 17q25.3. Three index cases of pedigrees 5, 11 and 18 were analyzed. The blue lines represent the copy numbers (log 2 ratio) averaged over 10 SNPs. The copy numbers were compared with control spouse of 2 of pedigree 18. (TIF) [file pone.0022542.s006.tif]

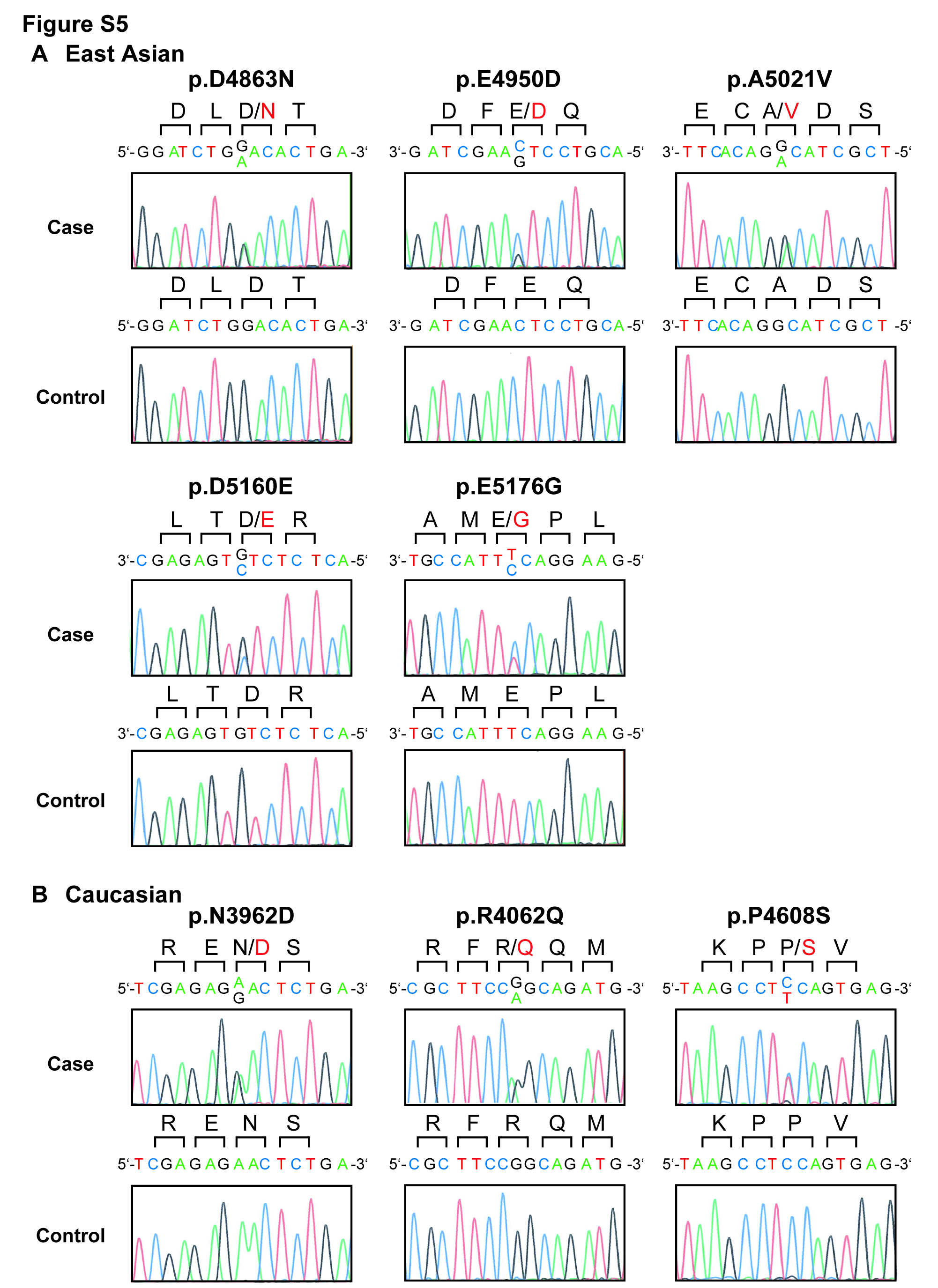

Supplement: Figure S5 — Sequence chromatograms for the eight novel variants. (TIF) [file pone.0022542.s007.tif]

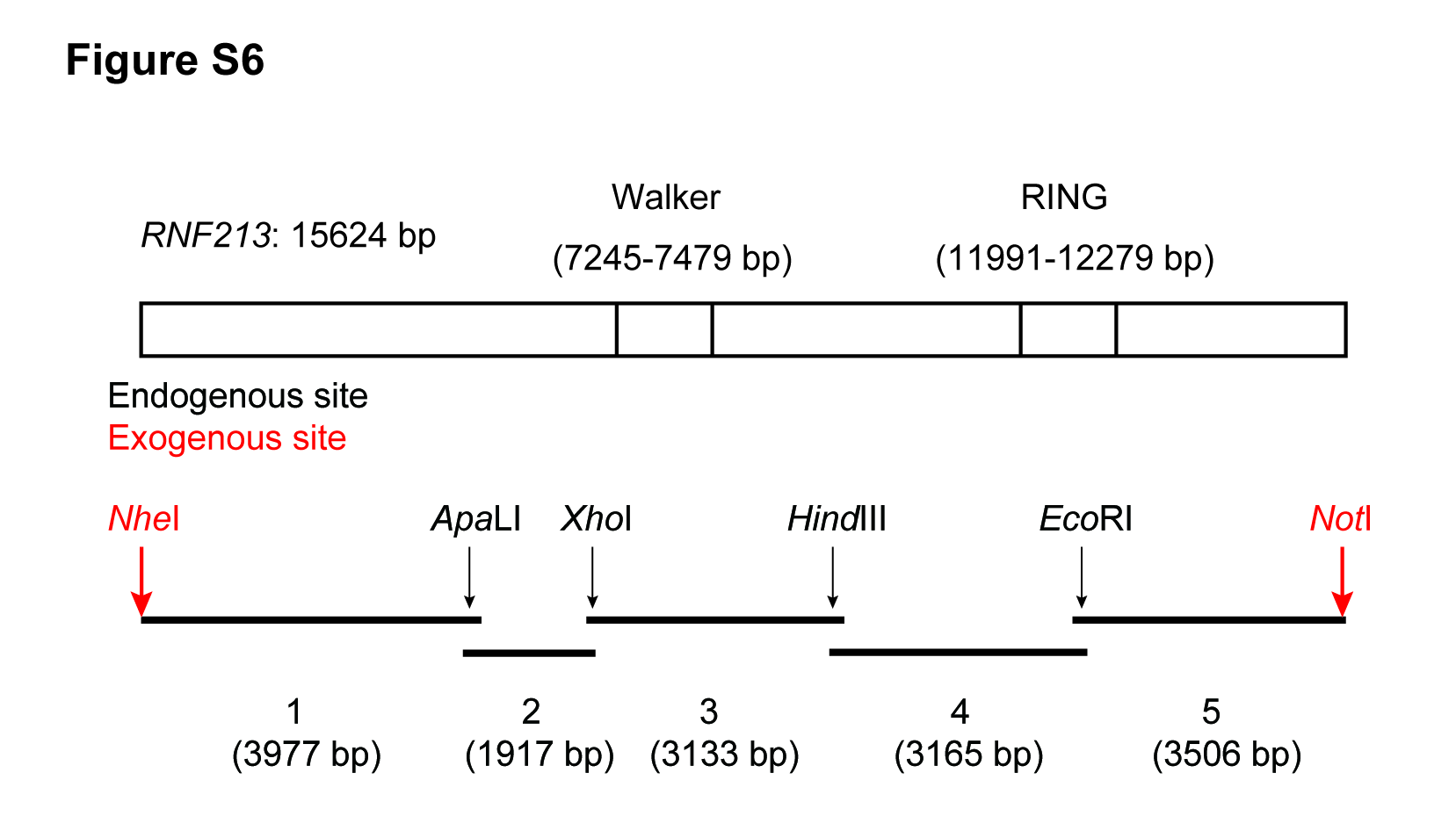

Supplement: Figure S6 — Cloning of full-length RNF213 cDNA. Schematic representation of the internal connecting site of RNF213 cDNA. RT-PCR with primers set on RNF213 resulted in amplification of the expected fragments. First, fragments 1, 2, 4, and 5 were cloned into a pcDNA3.1+ vector. Second, fragment 3 was cloned into the vector carrying fragment 2, and fragment 5 was subcloned into the vector carrying fragment 4. Then fragments 2–3 and 4–5 were subcloned into fragment 1 using the restriction enzyme sites indicated. (TIF) [file pone.0022542.s008.tif]

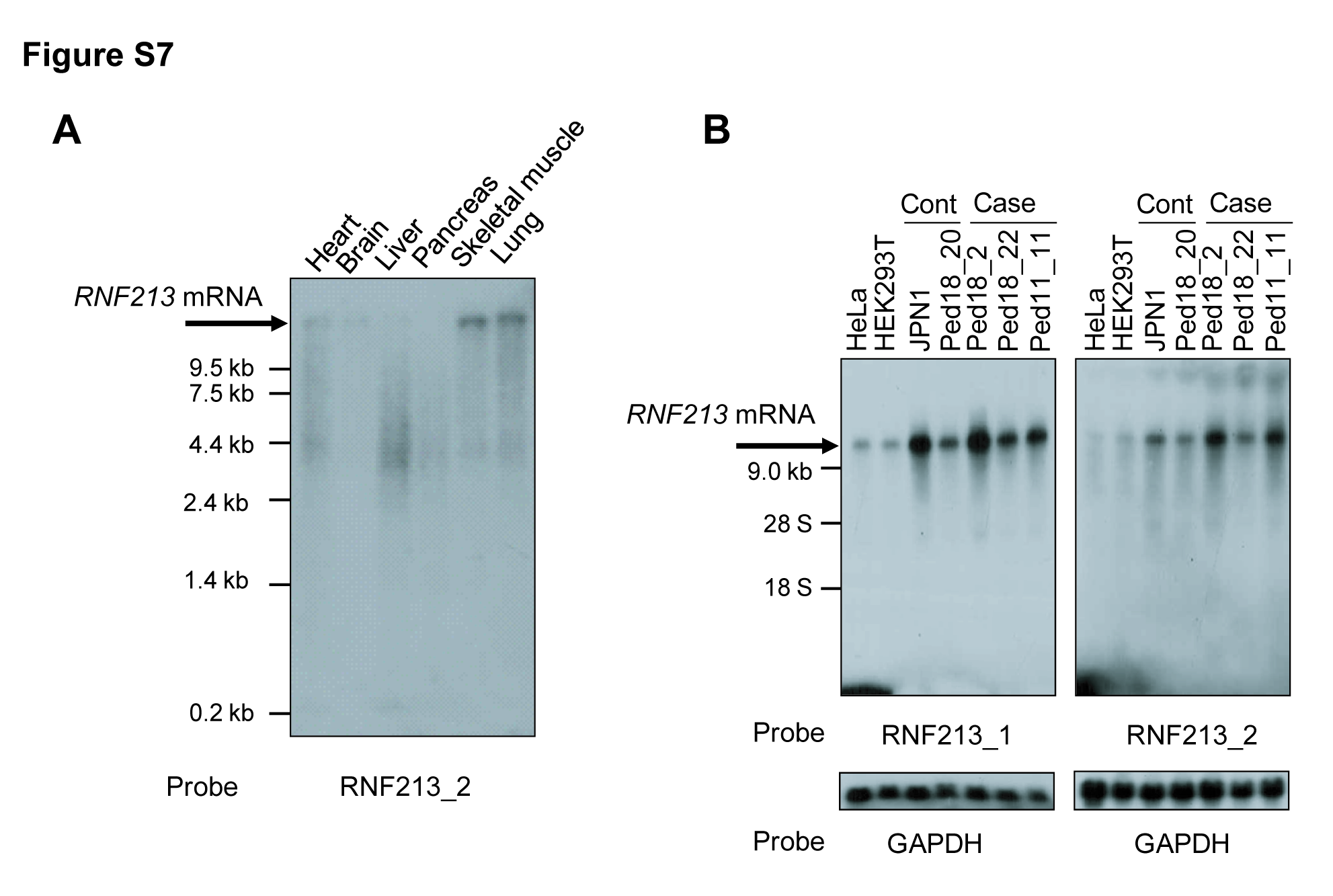

Supplement: Figure S7 — Northern blotting analysis of RNF213 mRNA. (A) RNF213 mRNA expression in the indicated human tissues (heart, brain, liver, pancreas, skeletal muscle, and lung). Arrow indicates RNF213 mRNA. (B) RNF213 mRNA expression in cultured human cells (HeLa, HEK293T and LCLs (control: JPN1, Ped18_20 (a spouse of 2) and case: Ped18_2, Ped18_22, Ped11_11)) using radiolabelled probes corresponding to RNF213 coding regions. GAPDH mRNA expression is shown as a loading control. The position of RNA Millennium markers (Ambion) and positions of the 18S and 28S ribosomal RNAs are indicated on the left. (TIF) [file pone.0022542.s009.tif]

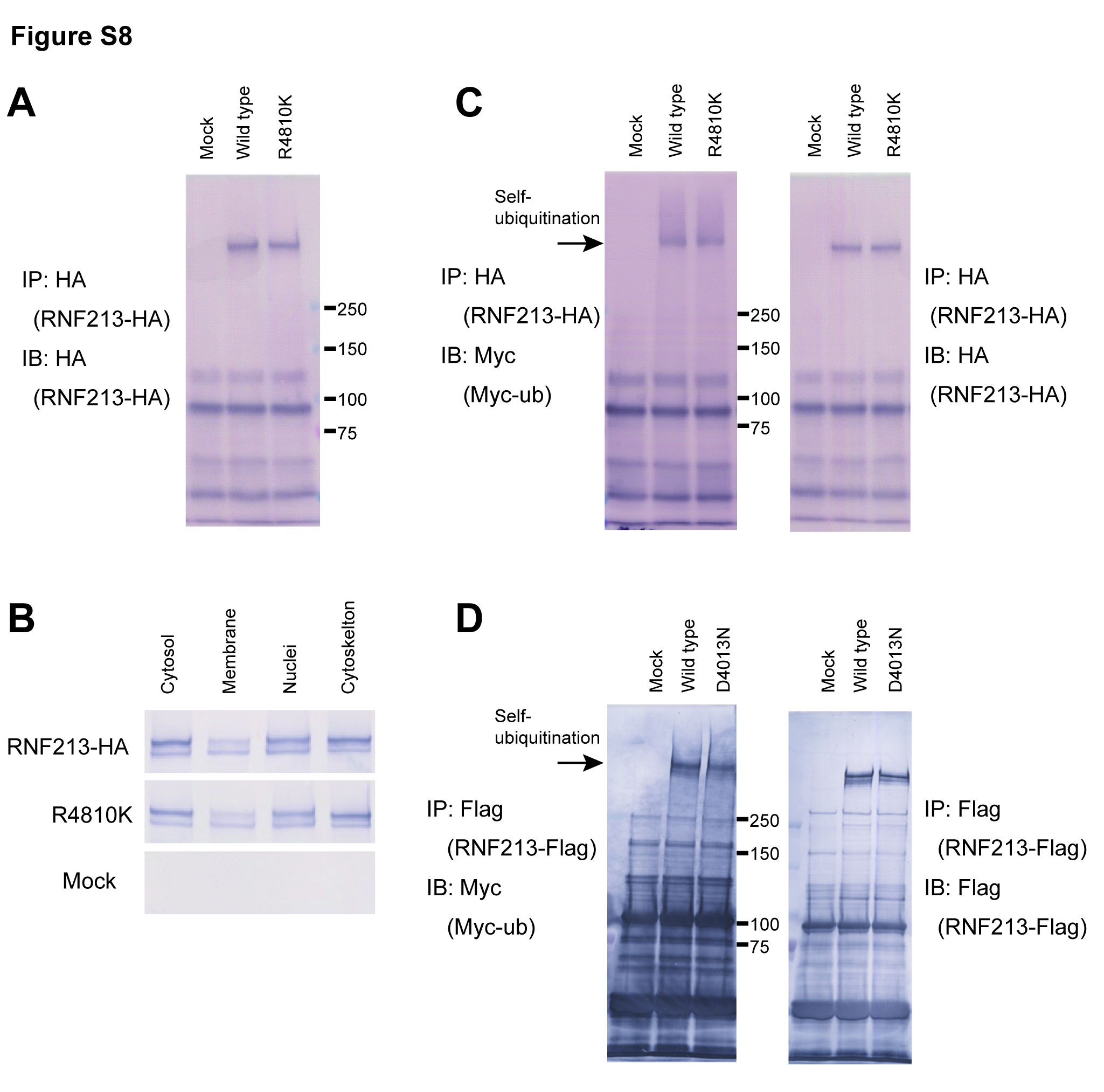

Supplement: Figure S8 — Characterization of the p.R4810K and p.D4013N allele proteins of RNF213. (A) Stability of the p.R4810K variant. Wild-type or p.R4810K variant of RNF213-HA were transiently expressed in HEK293 cells. Cells were lysed and subjected to immunoblotting with an anti-HA antibody. IP, immunoprecipitation. IB, immunoblot. (B) Subcellular localization of the p.R4810K variant of RNF213. HEK293 cells transiently expressing the wild-type or p.R4810K variant of RNF213-HA were fractionated into cytosol, membrane/organelle, nucleus, and cytoskeleton using different lysis reagents (ProteoExtract kit, Calbiochem). (C) Self-ubiquitination of the p.R4810K variant of RNF213. HEK293 cells transiently expressing the wild-type or R4810K variant of RNF213-HA and Myc-ubiquitin were lysed and subjected to immunoprecipitation using an anti-HA antibody, followed by immunoblotting using an anti-Myc antibody. (D) Self-ubiquitination of the p.D4013N mutant of RNF213. HEK293 cells transiently expressing the wild-type or p.D4013N mutant of RNF213-Flag and Myc-ubiquitin were lysed and subjected to immunoprecipitation using an anti-Flag antibody, followed by immunoblotting using an anti-Myc antibody. (TIF) [file pone.0022542.s010.tif]

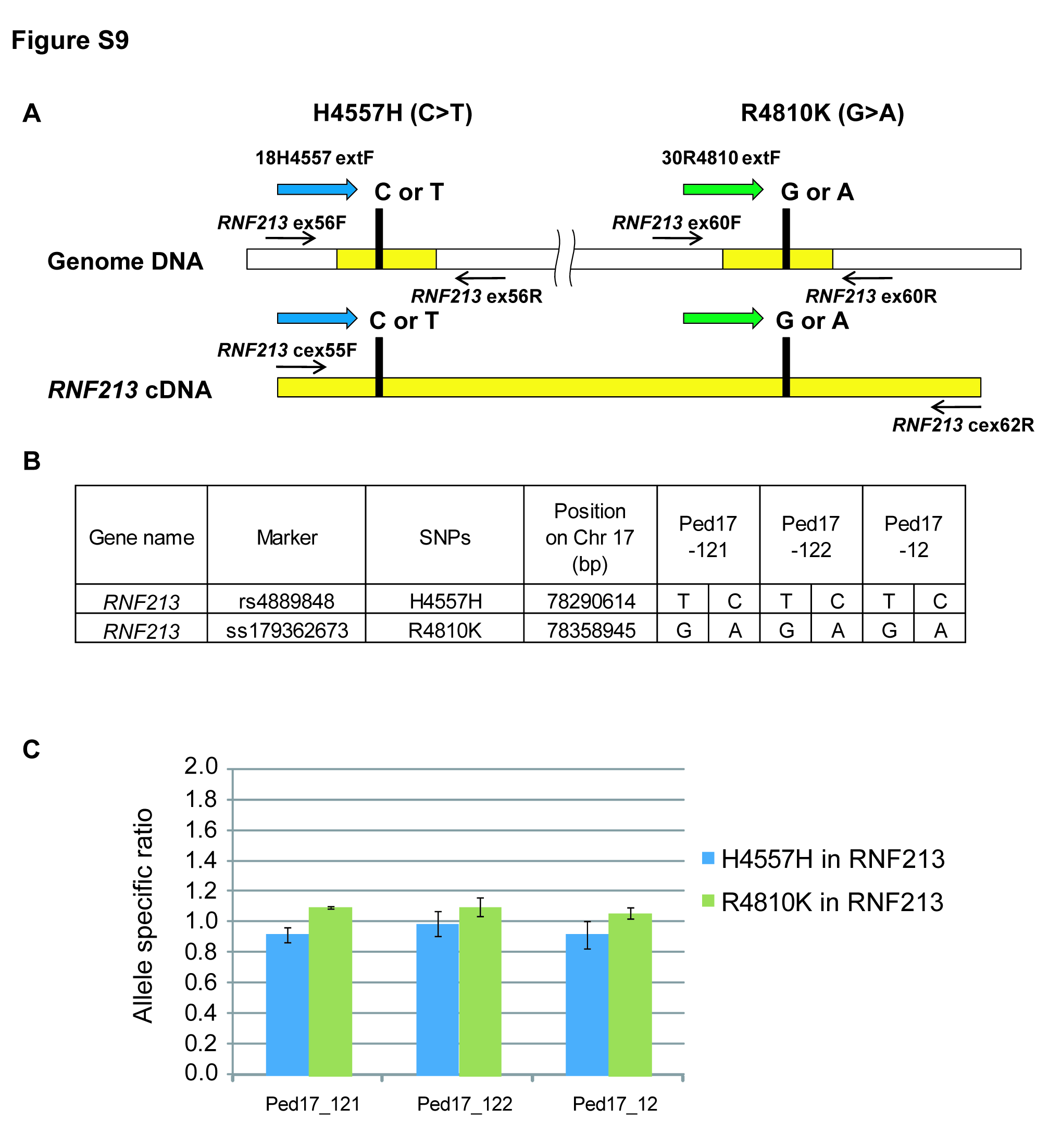

Supplement: Figure S9 — Allele-specific mRNA expression of RNF213 by labeling and detection of the two alleles of marker SNPs p.R4810K and p.H4557H. (A) Design of allele-specific mRNA expression using SNaPshot assay. Arrows indicate primer positions for amplification. Bold arrows indicate extension primers for each SNP. (B) Genotypes of RNF213 gene at two SNPs (p.H4557H and p.R4810K) in cases for SNaPshot assay. (C) Allele-specific ratio of RNF213 mRNA expression in LCLs from SNP heterozygous patients. The common allele/rare allele ratio from cDNA was normalized to that ratio from genomic DNA of the same individual. Data are shown as means ± S.D. of three independent experiments. There was no statistically significant difference between the two alleles for each SNP. Significance was tested by Student's t-test. A p<0.05 was considered to be significant. (TIF) [file pone.0022542.s011.tif]

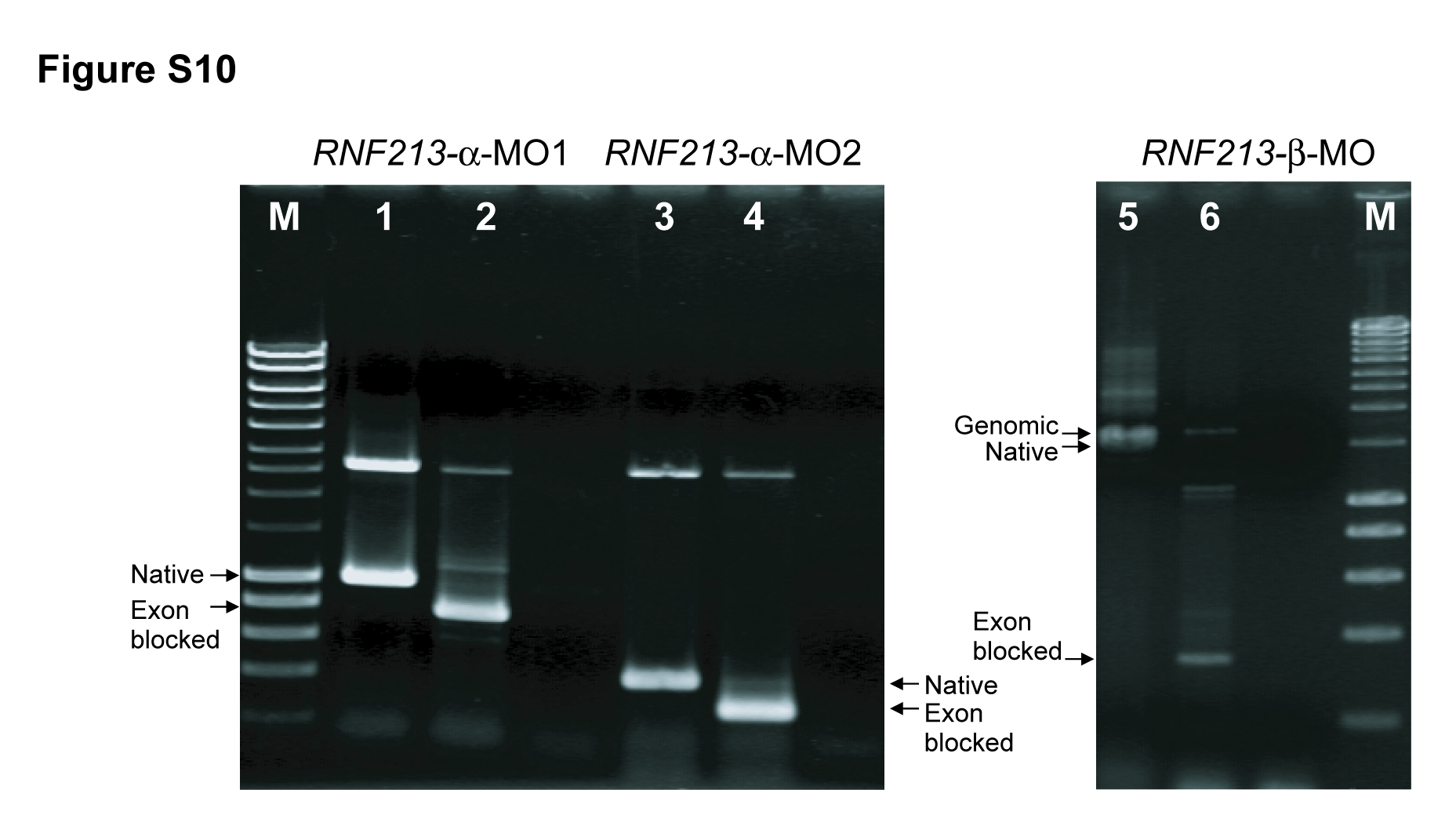

Supplement: Figure S10 — Splicing ablation of RNF213 transcripts by morpholino injection. RT-PCR showing the defective splicing induced by RNF213-α-MO1 and MO2 pairs and a RNF213-β-MO. Compared with the results of PCR (using primer pairs RNF213-α_2 and 2R, RNF213-β_3 and 3R, and RNF213-β_1 and 1R) from noninjected embryos, where a single band was generated (lanes 1, 3, and 5, marked “native”), split bands were detected in PCR using embryos injected with a RNF213-α-MO1, MO2 pair and a RNF213-β-MO (lanes 2, 4, and 6, marked “exon blocked”). M, 1-kb ladder DNA marker. Band indicated by ‘genomic’ is the amplified genomic sequence. Equal amounts of PCR products and marker were loaded in each lane. Intensity of RT-PCR products indicates that RNF213-α is dominantly expressed in vivo. (TIF) [file pone.0022542.s012.tif]

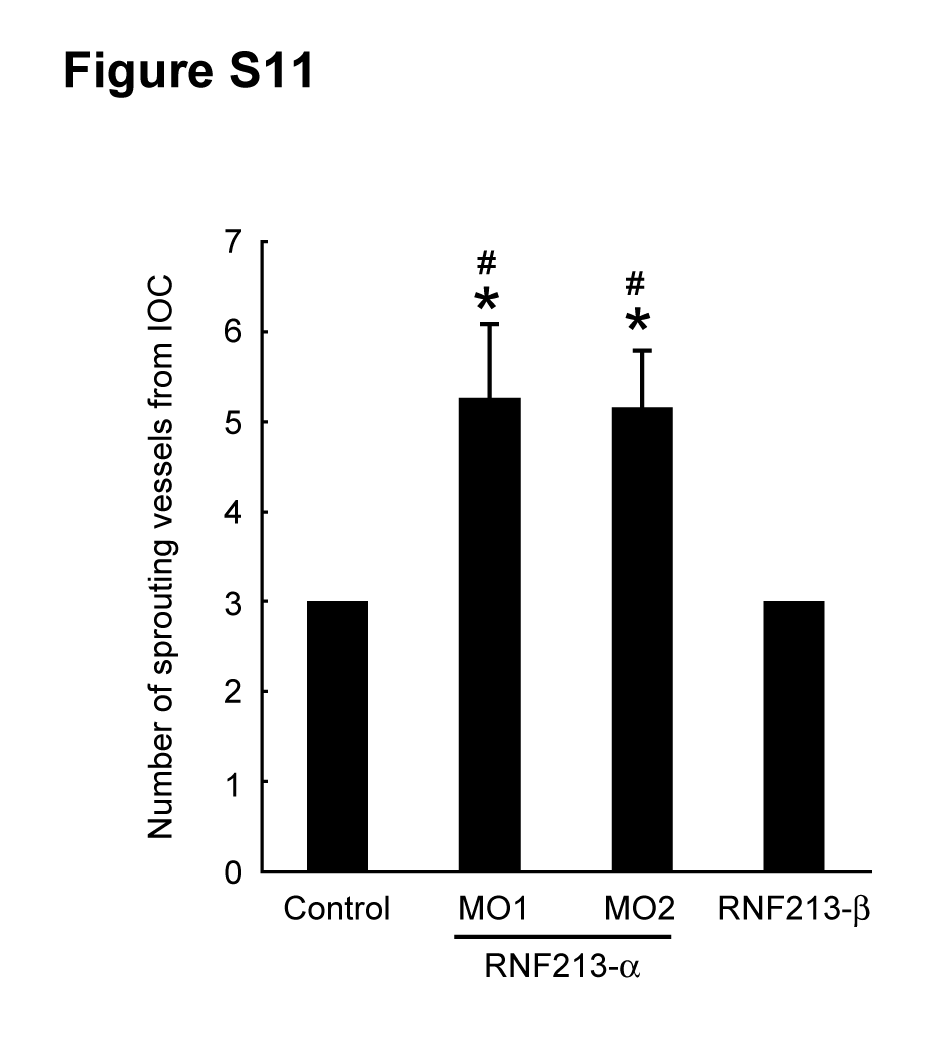

Supplement: Figure S11 — RNF213 morphants show multiple sprouting vessels from IOC. Bar graphs showing the number of sprouting vessels from IOC of Tg(fli-EGFP)y1 embryos 72 h post-fertilization. Each group was injected with 2.5 ng morpholinos (MO1, MO2) per embryo indicated in each lane. n = 20 per group. Values are means ± SD. *p<0.01 versus control scramble morphants. #p<0.01 versus RNF213-β-morphants. Neither group of controls nor RNF213-β showed any extra sprouting vessels. (TIF) [file pone.0022542.s013.tif]
